# The Intraocular Pressure under Deep versus Moderate Neuromuscular Blockade during Low-Pressure Robot Assisted Laparoscopic Radical Prostatectomy in a Randomized Trial

**DOI:** 10.1371/journal.pone.0135412

**Published:** 2015-08-28

**Authors:** Young-Chul Yoo, Na Young Kim, Seokyung Shin, Young Deuk Choi, Jung Hwa Hong, Chan Yun Kim, HeeJoon Park, Sun-Joon Bai

**Affiliations:** 1 Department of Anesthesiology and Pain Medicine, Anesthesia and Pain Research Institute, Yonsei University College of Medicine, Seoul, Republic of Korea; 2 Department of Urology, Urological Science Institute, Yonsei University College of Medicine, Seoul, Republic of Korea; 3 Biostatistics Collaboration Units, Department of Research Affairs, Yonsei University College of Medicine, Seoul, Republic of Korea; 4 Department of Ophthalmology, Institute of Vision Research, Yonsei University College of Medicine, Seoul, Republic of Korea; Uppsala University, SWEDEN

## Abstract

**Background:**

This study aimed to determine whether continuous deep neuromuscular blockade (NMB) improves the surgical conditions and facilitates robotic-assisted laparoscopic radical prostatectomy (RALRP) under low intra-abdominal pressure (IAP) to attenuate the increase in intraocular pressure (IOP) during CO_2_ pneumoperitoneum in the steep Trendelenburg (ST) position.

**Methods:**

Sixty-seven patients undergoing RALRP were randomly assigned to a moderate NMB group (Group M), including patients who received atracurium infusion until the end of the ST position, maintaining a train of four count of 1–2; and the deep NMB group (Group D), including patients who received rocuronium infusion, maintaining a post-tetanic count of 1–2. IOP was measured in all patients at nine separate time points. All RALRPs were performed by one surgeon, who rated the overall and worst surgical conditions at the end of the ST position.

**Results:**

The highest IOP value was observed at T4 (60 min after the ST position) in both Group M (23.3 ± 2.7 mmHg) and Group D (19.8 ± 2.1 mmHg). RALRP was accomplished at an IAP of 8 mmHg in 88% Group D patients and 25% Group M patients. The overall surgical condition grade was 4.0 (3.0–5.0) in Group D and 3.0 (2.0–5.0) in Group M (*P* < 0.001).

**Conclusion:**

The current study demonstrated that continuous deep NMB may improve surgical conditions and facilitate RALRP at a low IAP, resulting in significant attenuation of the increase on IOP. Moreover, low-pressure pneumoperitoneum, facilitated by deep NMB still provided acceptable surgical conditions.

**Trial Registration:**

ClinicalTrials.gov NCT02109133

## Introduction

Robotic-assisted laparoscopic radical prostatectomy (RALRP) is frequently used and advanced surgical technique in current practice.[1] Because RALRP has to be performed in a limited retroperitoneal space, insufflation of the abdomen with carbon dioxide (CO_2_) pneumoperitoneum and the steep Trendelenburg (ST) position are required to ensure a better surgical view.[2]

However, CO_2_ pneumoperitoneum at a high intra-abdominal pressure (IAP) combined with the ST position not only causes adverse hemodynamic effects [3] but also increases the intraocular pressure (IOP), which may result in severe ophthalmic damage such as ischemic optic neuropathy.[4,5] Despite its rare incidence, ophthalmic damage after RALRP could be the severe outcome. Therefore, the clinical importance of the prevention of IOP increase during RALRP cannot be emphasized enough.[6]

The European Association for Endoscopic Surgery recommends the use of the lowest IAP (rather than routine pressures) for adequate exposure of the surgical field.[7] Therefore, adequate working space should always be achieved with careful consideration of IAP for the patient’s safety.[8] Recent studies reported that deep neuromuscular blockade (NMB) improved surgical conditions,[9–11] even during low-pressure laparoscopic surgeries.[12–14] However, no study has evaluated the effects of the surgical conditions under deep NMB on IOP during RALRP.

Therefore, this study assessed the hypothesis that continuous deep NMB improves the surgical conditions and facilitates RALRP at a low IAP, which, in turn, attenuates the increase in IOP during CO_2_ pneumoperitoneum in the ST position. The level of surgeon satisfaction with the surgical conditions at a low IAP was also evaluated.

## Patients and Methods

The protocol for this trial and the CONSORT checklist are available as Supporting Information files (S1 CONSORT Checklist, S1 and S2 Protocols).

### Study population

This prospective, randomized, double blind trial was conducted at the Severance Hospital, Yonsei University Health System, Seoul, Republic of Korea between April 2014 and December 2014. Approval of study protocol was obtained from the Institutional Review Board and Hospital Research Ethics Committee of Severance Hospital, Yonsei University Health System on March 2014, and was subsequently registered at http://clinicaltrials.gov (registration number NCT02109133). Patients with an American Society of Anesthesiologist (ASA) grade of I or II and aged 50–80 years who were scheduled to undergo elective RALRP visited the Anesthesiology preoperative evaluation clinic, and were enrolled after they provided written informed consent. Patients who had undergone previous ophthalmic surgery or were taking medications for glaucoma, those with current ophthalmic disease (glaucoma, diabetic retinopathy, cataract, and retinal detachment), and those with a baseline IOP of >30 mmHg were excluded. Patients with a history of allergy to sugammadex or neuromuscular blocking agents, known or suspected neuromuscular diseases, past history of retroperitoneal surgery, hypersensitivity to anesthetic agents, uncontrolled hypertension, liver or kidney disease, previous or familial history of malignant hyperthermia, medications that interact with muscle relaxants (anticonvulsants, certain antibiotics, magnesium, etc.), a body mass index (BMI) of 30 kg/m^2^, neurological or psychiatric illness, and mental retardation, as well as those incapable of reading the consent form because of illiteracy or language barriers, were excluded.

### Randomization and Allocation

**Fig 1:**
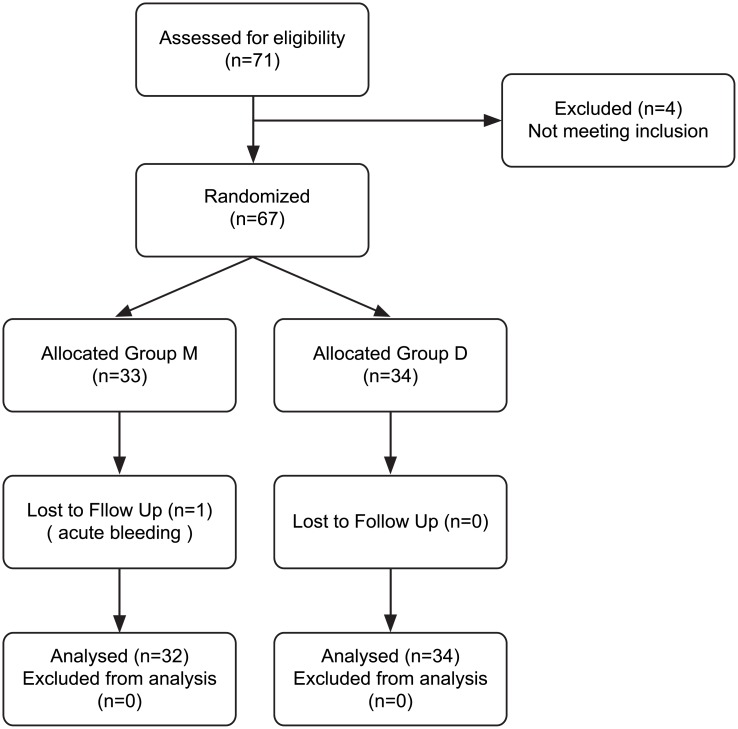
CONSORT Flow Diagram. Group M, moderate NMB group; Group D, deep NMB group.

After enrollment, patients were randomly allocated to either deep NMB Group (Group D, n = 34) or moderate NMB group (Group M, n = 33) according to predetermined randomization sequence, which was generated in www.random.org with no dividing blocks and was covered up in a sealed envelope. An investigator who did not participate in IOP measurements and care of the enrolled patients performed patients’ enrollment and randomization, and prepared all study medications according to the group allocation. The attending anesthetist who managed the patients in the operating room was also not blinded. However, the ophthalmologic assessor, patients, surgical team, research team, and anesthetists who managed the patients in the recovery room were blinded to group allocation. The CONSORT Flow Diagram is reported in Fig 1.

### Perioperative Protocol

Patients were administered 0.05 mg/kg of intramuscular midazolam as premedication. On arrival in the operating room, routine monitoring of noninvasive arterial blood pressure, electrocardiogram (ECG), oxygen saturation (SpO_2_), and bispectral index (BIS) (Aspect A-2000; Aspect Medical System Inc., Newton, MA) were applied to the patient. Following the induction of general anesthesia with propofol (2 mg/kg) and remifentanil infusion (0.05–0.1 μg/kg/min), the radial artery was catheterized for continuous monitoring of arterial blood pressure and repetition of blood gas analysis.

Mechanical ventilation was applied with a tidal volume of 8 mL/kg ideal body weight in 50% oxygen with air, a positive end-expiratory pressure of 5 cmH_2_O, and an inspiratory time:expiratory time ratio of 1:2. The respiratory rate was adjusted to 10–20 breaths/min to maintain the end-tidal CO_2_ tension (ETCO_2_) at 35–42 mmHg. The maintenance of anesthesia was undergone with sevoflurane (0.6–2.3 age-adjusted minimal alveolar concentration) and remifentanil (0.03–0.1 μg/kg/min) to target BIS scores of 40 to 60. Neuromuscular monitoring was performed using accelomyography (TOF-Watch SX, Organon Ltd, Ireland) of the corrugator supercilli (CS) muscle. NMB agents (rocuronium or atracurium) were administered following calibration and stabilization of the train of four (TOF)-Watch.

The patients were randomly allocated to one of two groups. Group D (deep NMB group) included patients who received an intravenous (IV) rocuronium bolus (1.0 mg/kg) following the continuous infusion of 0.6 mg/kg until the end of the ST position. Dose titration was assigned to an attending anesthetist via regulation of the bolus infusion speed to maintain a post-tetanic count (PTC) of 1 to 2. Sugammadex was administered to reverse the effects of NMB after surgery. Group M (moderate NMB group) included patients who received an IV atracurium bolus (0.5 mg/kg) following the continuous infusion of 0.3 mg/kg until the end of the ST position. Dose titration was assigned to an attending anesthetist via regulation of the bolus infusion speed to maintain a TOF count of 1 to 2. Neostigmine was used to reverse the effects of NMB after surgery. TOF was assessed every 15 min, and PTC was assessed if TOF was 0. Pneumoperitoneum was induced with CO_2_ insufflation of 20 mmHg. Following the insertion of trocars, an IAP of 8 mmHg was set from the previous 20 mmHg, and a remote control was used to place the patients in a precise 29° Trendelenburg position. All RALRP procedures were performed by a single experienced surgeon (Y.D.C) who was blinded to the group allocation.

The following steps were implemented in the event of insufficient surgical conditions in both groups; the decision of the insufficient surgical condition was done by the surgeon.

**Fig 2:**
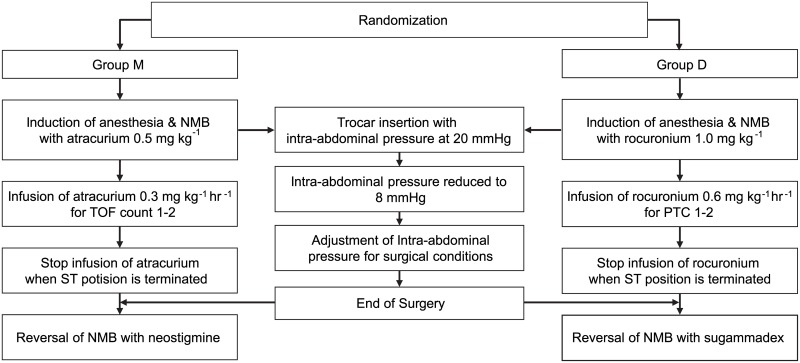
Study protocol. NMB, neuromuscular block; Group M, moderate NMB group; Group D, deep NMB group; TOF, train of four; PTC, post-tetanic count; ST, steep Trendelenburg.

1. Increase in IAP to 10 mmHg
2. If still insufficient, increase in IAP to 12 mmHg
3. If still insufficient, increase in IAP to 15 mmHg
4. If still insufficient, increase in IAP to 20 mmHg
5. If still insufficient, drop out (Fig 2)

Topical anesthetic eye drops (0.5% proparacaine HCl; Alcon, Seoul, Korea) were given to the patients in both groups. One blinded ophthalmologist measured IOP in all patients, three times at nine separate time points (Table 1), using the Tono-Pen XL handheld tonometer (Medtronic, Jacksonville, FL). The median value of the three IOP measurements was analyzed for the data.

**Table 1:** Time points of intraocular pressure measurement.

Time point	Event
T0	Before anesthesia induction (patient awake in the supine position)
T1	5 min after anesthesia induction (patient mechanically ventilated, before CO_2_ pneumoperitoneum in the supine position)
T2	5 min after establishing CO_2_ pneumoperitoneum in the horizontal position
T3	30min after CO_2_ pneumoperitoneum in the ST position
T4	60 min after CO_2_ pneumoperitoneum in the ST position
T5	5 min after returning to the horizontal position with CO_2_ desufflation
T6	5 min after tracheal extubation in the operating room
T7	30 min after tracheal extubation in the recovery room
T8	60 min after tracheal extubation in the recovery room

ST: steep Trendelenburg

### Study endpoints

The primary endpoint was to compare the maximum IOP after being positioned in the ST position under CO_2_ pneumoperitoneum between the two groups. The secondary endpoints were the surgical condition ratings given by the surgeon, comparison of the overall trends in IAP changes, and correlation between IOP and IAP during pneumoperitoneum in both groups. At the end of the ST position, the surgeon was asked to rate the overall surgical conditions and worst surgical conditions using the 5-point rating scale as previously described [12]: Grade 5 (optimal), optimal surgical conditions; grade 4 (good), nonoptimal conditions, but an intervention is not required; grade 3 (acceptable), wide surgical view, but an intervention can improve surgical conditions, grade 2 (poor), inadequate conditions, there is a visible view, but an intervention is necessary to ensure acceptable surgical conditions; grade 1 (extremely poor), inability to perform surgery; therefore, intervention is necessary.

NMB was maintained from induction until the end of the ST position. The patient was extubated only after regaining consciousness and exhibiting a TOF ratio of > 0.9. After extubation, the patients were monitored for a minimum of 60 min in the post-anesthetic care unit (PACU). In addition, postoperative pain was assessed using a verbal Numerical Rating Scale for pain (vNRS, 0 = no pain and 10 = worst pain imaginable) by blinded recovery nurses. Any postoperative respiratory events or known unfavorable events such as hypotension, dry mouth, nausea and vomiting, abdominal discomfort, headache, bradycardia, and dizziness were monitored.

### Sample Size and Statistical Analysis

In a previous study [5], IOP under pneumoperitoneum in the Trendelenberg position was 19.9 ± 3.8 mmHg in a propofol-based total intravenous (TIVA) group and 23.5 ± 4.3 mmHg in a sevoflurane inhalational anesthesia group. To detect a 3.6-mmHg difference in IOP, power estimation analysis suggested that 31 patients per group would be required to obtain a power of 90%, considering a type I error of 0.05. Considering a drop-out rate of 10%, we recruited 34 patients in each group.

All statistical analyses were performed using SAS software version 9.2 (SAS Inc., Cary, NC, USA) and IBM SPSS Statistics 20 (SPSS Inc., Chicago, IL, USA). All continuous values were shown as mean ± SD or median (range), and the number of patients (%) was used for all categorical values. Between-group comparisons of continuous variables were performed by independent Student’s *t*-test. Repeated measured variables such as IOP, MBP, ETCO_2_, and PIP were analyzed using a linear mixed model with the patient indicator as a random effect and group, time, and group-by-time as fixed effects. When the interaction of group, time, and group-by-time showed statistical significance, post hoc analysis was performed with Bonferroni correction for the adjustment for multiple comparisons. Analysis was performed using a univariate linear mixed model to identify possible predictors of alteration in IOP over the T3–T4 period. Multivariate linear mixed model analysis was performed using variables that were statistically significant in the univariate model at the 0.05 level. All statistical tests were two-tailed, and *P*-values of < 0.05 were considered statistically significant.

## Results

For eligibility, a total of 71 patients were screened, and four patients who did not meet the inclusion criteria because of two previous cataract surgeries, current glaucoma with medication, and obesity (BMI > 30 kg/m^2^). Finally 67 patients were randomly assigned to the two groups. One patient in Group M was dropped out because of acute uncontrolled bleeding during surgery. Consequently, the remaining 66 patients (34 in Group D and 32 in Group M) were analyzed, with no missing data (Fig 1).

The characteristics of the patients were comparable between groups (Table 2). The duration of anesthesia, surgery, pneumoperitoneum, and Trendelenburg were also similar between groups. Apart from the total amount of CO_2_, there were no significant differences in the amount of ephedrine, fluid intake, blood loss, and urine output. The total CO_2_ amount was significantly lower in Group D (407 ± 228 L) than in Group M (593 ± 248 L) (*P* = 0.003). Patients in Group D showed a TOF count of 0 and an average PTC of 1.6 ± 0.6, while those in Group M showed a TOF count of 1.7 ± 0.3 (Table 3). The overall and worst surgical condition grades are also shown in Table 3. There were significant differences in ratings between the two groups. The overall surgical condition grade was 4.0 (3.0–5.0) in Group D and 3.0 (2.0–5.0) in Group M, while the worst surgical condition grade was 4.0 (2.0–5.0) in Group D and 2.0 (2.0–4.0) in Group M (*P* < 0.001).

**Table 2:** Patient characteristics.

Variables	Group M (n = 32)	Group D (n = 34)
Age (years)	63.9 ± 6.1	61.5 ± 5.4
Height (cm)	167.8 ± 4.7	168.9 ± 4.4
Weight (kg)	68.8 ± 9.0	67.3 ±
BMI (kg/m^2^)	24.4 ± 2.5	23.6 ± 2.0
ASA class I/II	8/24	13/21
Hypertension	14 (44)	13 (38)
Diabetes mellitus	3 (9)	4 (12)
Asthma	0 (0)	1 (3)

Values are mean ± SD, numbers (%); BMI, body mass index; ASA, American Society of Anesthesiologists; Group M, moderate neuromuscular blockade group; Group D, deep neuromuscular blockade group

**Table 3:** Intraoperative variables.

Variables	Group M (n = 32)	Group D (n = 34)	P value
Duration, anesthesia (min)	160 ± 31	156 ± 25	0.484
Duration, operation (min)	115 ± 32	111 ± 21	0.583
Duration, pneumoperitoneum (min)	83 ± 25	80 ± 18	0.202
Duration, Trendelenburg (min)	68 ± 18	69 ± 17	0.801
Total CO_2_ amount (L)	592 ± 248	407 ± 228	0.003*
Total ephedrine amount (mg)	4.0 ± 6.6	4.0 ± 4.4	< 0.001*
Total fluid intake (mL)	1210 ± 393	1316 ± 325	0.235
Total blood loss (mL)	467 ± 344	416 ± 235	0.482
Total urine output (mL)	199 ± 119	247 ± 114	0.101
Overall surgical condition	3 (2–5)	4 (3–5)	< 0.001*
Worst surgical condition	2 (2–4)	4 (2–5)	< 0.001*
Total atracurium amounts (mg)	69 ± 20		
Total rocuronium amounts (mg)		209 ± 37	
TOF	1.7 ± 0.3		
PTC		1.6 ± 0.6	

Values are mean ± SD, numbers (%), and median (minimum—maximum); TOF, train of four; PTC, post-tetanic count;Group M, moderate neuromuscular blockade group; Group D, deep neuromuscular blockade group.

* *P* < 0.05

**Fig 3:**
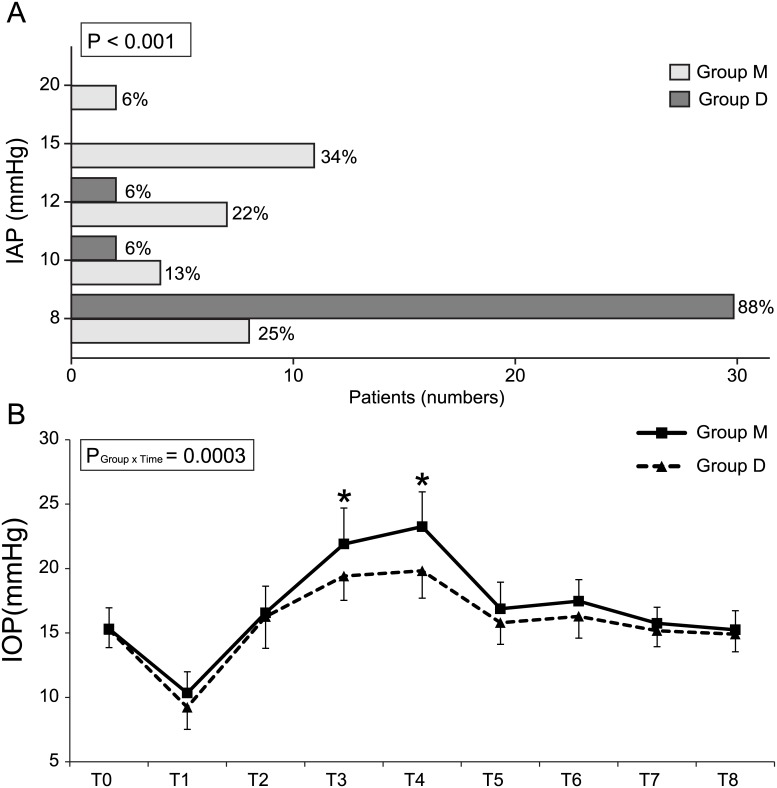
Distribution of intra-abdominal pressure (IAP) during RALRP (A) and the mean intraocular pressure (IOP) (B). Group M, moderate neuromuscular blockade group; Group D, deep neuromuscular blockade group; RALRP, robotic-assisted laparoscopic radical prostatectomy. * P < 0.05 compared to Group M.

RALRP was accomplished at an IAP of 8 mmHg in 30 (88%) patients of Group D compared with 8 (25%) patients of Group M in 8mmHg of pneumoperitoneum. IAP was elevated to 10 or 12 mmHg in the remaining 4 (12%) patients of Group D and up to 20mmHg in 2 (6%) patients of Group M, respectively, due to insufficient surgical conditions (Fig 3A). There were no differences in baseline IOP between the two groups. There was no patient who had baseline IOPs higher than 20 mmHg. The analysis with linear mixed model was shown that there was a significant intergroup difference in IOP over time (*P* = 0.0003) (Fig 3B). Even after post hoc analysis with Bonferonni correction, IOP at T3 and T4 during pneumoperitoneum in the ST position were significantly lower in Group D than in Group M (*P* < 0.001).

**Fig 4:**
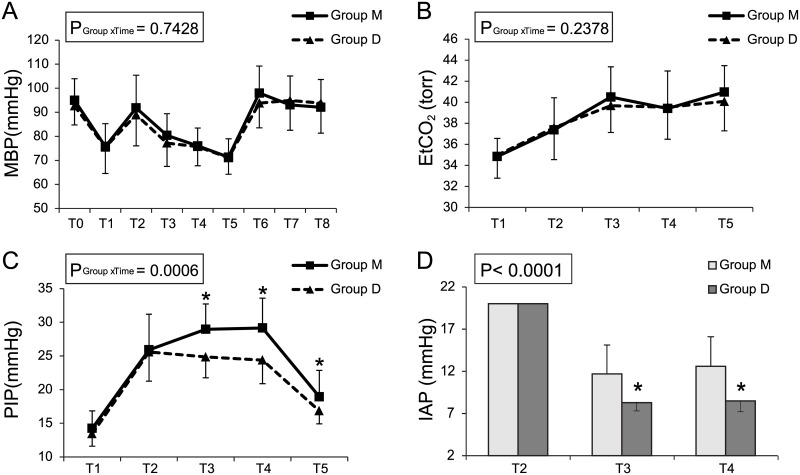
Mean blood pressure (MBP) (A), end-tidal CO_2_ (ETCO_2_) (B), peak inspiratory pressure (PIP) (C), and intra-abdominal pressure (IAP) (D) in Group M and Group D. Group M, moderate neuromuscular blockade group; Group D, deep neuromuscular blockade group. * P < 0.05 compared to Group M.

Except for PIP, there were no significant differences in MBP and EtCO_2_ between the two groups (Fig 4A and 4B). Fig 4C shows that there was a significant difference in PIP over time between the two groups through the linear mixed model analysis (*P* = 0.0006). Post hoc analysis with Bonferonni correction indicated that PIP at T2, T3, and T4 were significantly lower in Group D than in Group M (*P* < 0.001, *P* < 0.001, *P* = 0.0375, respectively). In addition, IAP at T3 and T4 showed significant differences between the two groups (*P* < 0.001) (Fig 4D).

As shown in Table 4, there were no significant differences in the postoperative respiratory variables, with the exception of SpO_2_ and degree of dry mouth (Table 4). Vomiting did not occur in any of the patients, while nausea occurred in 4 patients in Group M and 1 patient in Group D, with no significant difference. Postoperative pain as assessed by v-NRS was also comparable between groups. Finally, there was no patient who suffered from ocular complications and hemodynamic instability.

**Table 4:** Postoperative recovery variables.

Variables	Group M (n = 32)	Group D (n = 34)	P value
Sugammadex (mg)		158 ± 57	
Neostigmine (mg)	1 ± 0		
Time in PACU (min)	75 ± 51	61 ± 4	0.126
SpO_2_ (%)	99.1 ± 1.0	99.5 ± 1.0	0.032*
Respiratory rate	14.7 ± 1.9	14.9 ± 2.1	0.693
VNRS in PACU	2.97 ± 1.42	2.62 ± 0.70	0.204
Nausea	4 (13)	1 (3)	0.142
Dry mouth	1.4 ± 1.0	0.9 ± 0.9	0.021*

Values are mean ± SD, numbers (%); PACU, postanesthetic care unit; SpO_2_, oxygen saturation; VNRS, verbal numerical rating scale; Group M, moderate neuromuscular blockade group; Group D, deep neuromuscular blockade group.

* *P* < 0.05

## Discussion

The current study demonstrated that continuous deep NMB may facilitate RALRP under a low IAP, resulting in improvement of surgical conditions and significant attenuation of the IOP increase during CO_2_ pneumoperitoneum in the ST position. Furthermore, low pressure pneumoperitoneum, facilitated by deep NMB still provided satisfactory surgical conditions.

In order to have adequate working space for optimal surgical view and no difficulty in laparoscopic device handling, CO_2_ pneumoperitoneum is required during laparoscopic surgery.[15–17] Furthermore, RALRP requires the ST position and, frequently, a high insufflation pressure for optimal surgical view, which causes an increase in IOP.[4,5] Also, the majority of patients undergoing RALRP are older with various comorbidities, which will leave the patient prone to the consequent ocular damage due to the increased IOP. [18–20]

Several factors affect the working space during laparoscopic surgery.[8] It is commonly believed that a higher pneumoperitonuem pressure results in better exposure of the surgical field.[21] However, the increase in pressure at a low IAP offers a better working space compared with that at a high IAP.[8] This is because the compliance of the abdominal wall decreases at a high IAP.[22,23] From this perspective, several studies regarding the feasibility and advantages of low-pressure laparoscopic surgery have been conducted.[24–27] Recently, studies reported that deep NMB helps in improving the quality of the surgical view,[9–11] even during low-pressure laparoscopic surgeries.[12–14] However, to the best of our knowledge, this is the first study regarding the influence of the surgical conditions under continuous deep NMB on IOP in the ST position under pneumoperitoneum during RALRP.

A significantly greater number of procedures were successfully completed in Group D (88%) than in Group M (25%) at 8 mmHg, with the success rate in Group D being similar to that in previous reports (about 70% to 100%) comparing postoperative pain between low-pressure and standard-pressure laparoscopic surgeries.[24,26] However, in Group M, 75% patients showed an IAP increase adjustment. The average mean (SD) IAPs at T3 and T4 were 11.7 ± 3.4 and 12.6 ± 3.5 in Group M and 8.3 ± 1.0 and 8.5 ± 1.3 in Group D, respectively, which were lower than those used (15–18 mmHg) during conventional RALRP.[3,5,28] (Fig 4D).

In the current study, the increase in IOP at T3 and T4 (during pneumoperitoneum in the ST position) was significantly lower in Group D than in Group M, with the highest mean IOP observed at T4; 23.3 ± 2.7 mmHg in Group M and 19.8 ± 2.1 mmHg in Group D [difference, 3.43 mmHg; 95% confidence interval (CI), 2.24–4.62; *P* < 0.001]. Multiple factors affect the increase in IOP during pneumoperitoneum in the ST position: EtCO_2_, CVP, MBP, PIP, transperitoneal absorption of CO_2_, and duration of surgery.[4,5] However, in the present study, based on the univariate linear regression during T3 and T4, BMI, PIP, total CO_2_ amount, and IAP were significant predictors of IOP changes over the T3–T4 period. All these significant predictors were positively correlated (positive slope coefficients) with IOP and were included in multivariate models for the T3–T4 period. Multivariate analysis revealed that the only significant predictor of IOP was IAP (slope coefficient, 0.5178; *P* < 0.0001). A possible explanation may be that a low IAP led to a decrease in the peritoneal CO_2_ absorption and PIP, which may have attenuated the IOP increase.

All RALRP procedures were performed by a single surgeon with ample experience. Before this study, he had performed RALRP on 20 mmHg of pneumoperitoneum pressure during trocar insertion, and after the ST position, the pressure was decreased to 15 mmHg. However, IAP would be adjusted up to 20 mmHg in the event of uncontrolled bleeding or inadequate surgical conditions. Even though a new trial was required for this study, there were no significant differences between the first 34 and the last 34 procedures.

In regards to depth of NMB, the monitoring of adductor pollicis (AP) muscle has been applied as a validated method.[29,30] However, in the present study, the patients’ arm lay alongside the torso, and was covered by a surgical drape to prevent interference with the robotic arms. As an alternative method, the CS muscle offers a better reflection of the larynx and diaphragm.[31,32] Therefore, we measured the depth of NMB at the CS muscle instead of the AP muscle. This could be a limitation of our study. When measuring the depth of NMB at the CS muscle using acceleromyography, concomitant signals from the orbicularis oculi are inevitable, resulting in a mixed signal.[31] In addition, higher current is thought to be required to provoke maximal facial muscle contractions,[31] which could exacerbate the problem of direct muscle stimulation. However, we believe that the effect of this was minimized as a constant degree of NMB was maintained in each group.

The Other limitations of our study are as follows. It has been demonstrated that a low-pressure pneumoperitoneum can lead to a reduction in postoperative pain compared with a standard-pressure pneumoperitoneum.[24–27] In the current study however, there was no significant difference in postoperative pain between the two groups. This may be because IAP, even in Group M, was less than 12mmHg, which was defined as the standard pressure in previous studies.[24,25] Third, the scale used to evaluate the surgical conditions was more subjective compared with that used in previous studies.[12,14] We rated the overall and worst surgical conditions at the end of the ST position, whereas previous studies rated the surgical conditions at a regular time interval throughout the surgery. More objective and concrete measures are thus required for more precise results. Finally, in case of poor surgical conditions, no rescue doses of muscle relaxants or increased doses of general anesthetics was administered. Instead, IAP was increased from 8 mmHg to 10 mmHg to 12 mmHg to 15 mmHg to 20 mmHg (maximum) to improve the surgical conditions in both groups, whenever the surgeon felt uncomfortable or needed an adjustment during surgery. Although we assessed the depth of NMB every 15 min in both groups, there could have been occasions of inadequate NMB depth. Also, because a constant level of anesthesia was maintained, the effect of anesthesia depth on the surgical conditions was not evaluated.

Actually, With the exception of the surgeon’s satisfaction in this study, it is difficult to conclude that deep NMB is superior from an anaesthesiology point of view. However, considering the adverse effects of pneumoperitoneum and IOP increases in elderly patients undergoing RALRP, we believe that surgical conditions under low-pressure, facilitated by deep NMB may have clinical significance from both the anaesthetic and surgical perspective.

In conclusion, continuous deep NMB may lead to the improvement of surgical conditions and facilitate RALRP at low IAP levels, which may contribute to the significant attenuation of the IOP increase during pneumoperitoneum in the ST position. Further clinical studies are required for other types of surgeries to further clarify the effectiveness of deep NMB.

## Supporting Information

S1 CONSORT Checklist CONSORT Checklist. (DOC)

S1 File Sample case report form. (DOCX)

S2 File Sample informed consent form. (DOC)

S1 Protocol Clinical research protocols (English version). (DOCX)

S2 Protocol Clinical research protocols (original language version). (DOCX)
