# Correction: The Intraocular Pressure under Deep versus Moderate Neuromuscular Blockade during Low-Pressure Robot Assisted Laparoscopic Radical Prostatectomy in a Randomized Trial

**DOI:** 10.1371/journal.pone.0206339

**Published:** 2018-10-18

**Authors:** Young-Chul Yoo, Na Young Kim, Seokyung Shin, Young Deuk Choi, Jung Hwa Hong, Chan Yun Kim, HeeJoon Park, Sun-Joon Bai

The Corresponding Author’s contact information has changed. The correct email address for Sun-Joon Bai is: sjbae@yuhs.ac.

The underlying data for this study is omitted from the article. The authors have now provided the dataset. The underlying data can be viewed in S1 Dataset below.

## Supporting information

S1 Dataset (XLSX)

## References

[1] Yoo Y-C, Kim NY, Shin S, Choi YD, Hong JH, Kim CY, et al (2015) The Intraocular Pressure under Deep versus Moderate Neuromuscular Blockade during Low-Pressure Robot Assisted Laparoscopic Radical Prostatectomy in a Randomized Trial. PLoS ONE 10(8): e0135412 10.1371/journal.pone.0135412 26317357 PMC4552736

