# Supplementary material for: The Intraocular Pressure under Deep versus Moderate Neuromuscular Blockade during Low-Pressure Robot Assisted Laparoscopic Radical Prostatectomy in a Randomized Trial
Source: PLoS One. 2015 Aug 28;10(8):e0135412. doi: 10.1371/journal.pone.0135412 (PMC4552736; doi:10.1371/journal.pone.0135412)
Supplement: S1 File — (DOCX) [file pone.0135412.s002.docx]

**The impact of surgical validity through profound neuromuscular blockade on intraocular pressure in patients undergoing robot assisted laparoscopic radical prostatectomy**

**Date** ___________ **Inicial** ___________ **serial number** ___________ **Group** _______

**Sex/Age** ___ / ___ **Ht/Wt** ___ / ___ **BMI**______ **BSA**______ **ASA class** ______

**총 마취시간** ____________ **총** **수술시간** ___________

**PHx: HTN/DM** ( / ) **DM med** (PO / insulin ) **Others** ( )

**안과 질환 과거력** (녹내장/백내장/각막박리/당뇨성 망막증/안과적 수술여부_____________)

**Vasopressor 총 사용량**: ephedrine mg, phenylephrine mg **Postoperative discharge day:**

**Total muscle relaxant amount** : ___/___, **Total reverse amount** : ___/___

**Intraoperative variables**

|  | T0 | T1 | T2 | T3 | T4 | T5 | T6 | T7 | T8 |
| --- | --- | --- | --- | --- | --- | --- | --- | --- | --- |
| BIS |  |  |  |  |  |  |  |  |  |
| IOP | / / | / / | / / | / / | / / | / / | / / | / / | / / |
| Abd pr |  |  |  |  |  |  |  |  |  |
| **ventilator** | | | | | | |  |  |  |
| EtCO_2_ |  |  |  |  |  |  |  |  |  |
| M/V |  |  |  |  |  |  |  |  |  |
| TV |  |  |  |  |  |  |  |  |  |
| RR |  |  |  |  |  |  |  |  |  |
| PAP |  |  |  |  |  |  |  |  |  |
| Remifentanil |  |  |  |  |  |  |  |  |  |
| Agent |  |  |  |  |  |  |  |  |  |
| **vital** | | | | | | |  |  |  |
| SpO_2_ |  |  |  |  |  |  |  |  |  |
| BP |  |  |  |  |  |  |  |  |  |
| PR |  |  |  |  |  |  |  |  |  |
| **ABGA** | | | | | | |  |  |  |
| PH |  |  |  |  |  |  |  |  |  |
| PaO_2_ |  |  |  |  |  |  |  |  |  |
| PaCO_2_ |  |  |  |  |  |  |  |  |  |
| HCO3^-^ |  |  |  |  |  |  |  |  |  |
| Lactate |  |  |  |  |  |  |  |  |  |

**M/V**: minute volume, **PAP** : Peak airway pressure, **TV**: tital volume, **Abd pr**: abdominal pressure

**Total I & O**

| Fluid (crystalloid) |  |
| --- | --- |
| Fluid (Colloid) |  |
| Transfusion (PRBC) |  |
| Urine output |  |
| Bleeding |  |

| Time | Event |
| --- | --- |
| T0 | Before anesthesia induction (awake in supine, horizontal position) |
| T1 | 5 min after anesthesia induction (mechanically ventilated, before CO_2_ pneumoperitoneum in supine, horizontal position) |
| T2 | 5 min after establishing CO_2_ pneumoperitoneum in horizontal position |
| T3 | 30 min after CO_2_ pneumoperitoneum with steep Trendelenburg position |
| T4 | 60 min after CO_2_ pneumoperitoneum with steep Trendelenburg position |
| T5 | 5 min after returning to horizontal position with desufflation of CO_2_ |
| T6 | 5 min after tracheal extubation in the operating room |
| T7  T8 | 30 min after tracheal extubation in the recovery room  60 min after tracheal extubation in the recovery room |

**수술 중 surgical condition을 위하여 intra-abdominal pressure 를 조절한 시점과 정도**: pneumoperitoneum 후 ( ) min, ( ) mmHg

**전체 pneumoperitoneum 기간 중 intra-abdominal pressure 12 mmHg 이상이 필요했던 시간의 비율**: **전체 pneumoperitoneum 기간**:

**전체 trendelenbug position 기간**:

**전체 CO2 insufflation content (pneumoperitoneum 종료 시)** :

**Reversal agent 투여 이후 TOF ratio 0.9에 도달하는 시간** :

**Surgical condition rating**

| Worst surgical space condition |  |
| --- | --- |
| Overall surgical space condition |  |

**1: extremely poor, 2: poor, 3: acceptable, 4: good, 5: optimal**

Grade 5 (optimal), optimal surgical conditions; grade 4 (good), nonoptimal conditions, but an intervention is not required; grade 3 (acceptable), wide surgical view, but an intervention can improve surgical conditions, grade 2 (poor), inadequate conditions, there is a visible view, but an intervention is necessary to ensure acceptable surgical conditions; grade 1 (extremely poor), inability to perform surgery; therefore, intervention is necessary.

**회복실 입실 후 평가사항**

| **회복실 입실 중 평가 사항** | 30분 | 60분 |
| --- | --- | --- |
| **잔류근이완의 임상적 근거(예:호흡곤란)** |  |  |
| **근이완의재발생여부(예:TOF:0.7)** |  |  |
| **의식상태*** |  |  |
| **전신근육약화 *** |  |  |
| **5초 head tilt test (YES/NO)** |  |  |
| **오심 (none/mild/moderate/severe)** |  |  |
| **구토 (YES/NO)** |  |  |
| **구강건조 (none/mild/moderate/severe)** |  |  |

**의식상태*** awake and oriented, arousable with minimal stimulation, responsible only to tactile stimulation

**전신근육약화 *** 0-10 scale, 0: total paralysis, 10: normal muscle strength

**연구 책임자 성명_____________ 확인 서명_____________ 날짜 _______________**
