# Supplementary material for: The Intraocular Pressure under Deep versus Moderate Neuromuscular Blockade during Low-Pressure Robot Assisted Laparoscopic Radical Prostatectomy in a Randomized Trial
Source: PLoS One. 2015 Aug 28;10(8):e0135412. doi: 10.1371/journal.pone.0135412 (PMC4552736; doi:10.1371/journal.pone.0135412)
Supplement: S2 File — (DOC) [file pone.0135412.s003.doc]

**대상자 설명문**

**1. 연구제목**

다빈치 로봇을 이용한 복강경적 근치적 전립선 절제술을 시행 받는 환자에서 수술 중 깊은 근이완을 통한 수술 시야 확보가 안압의 변화에 미치는 영향 – 무작위 배정 연구 -

**2. 연구 배경 및 목적**

전립선 암은 미국 내에서 남자들에게 진단되는 암 중 가장 흔한 암중 하나로 서구 식생활을 따라가는 우리나라에서도 최근 급속도로 증가하고 있습니다. 전립선 암의 수술법은 여러 방법이 있지만 그 중 다빈치 로봇을 이용한 전립선 절제술은 가장 최신이며 기술적으로 가장 진보한 수술법 중 하나입니다. 다빈치 로봇을 이용한 전립선 절제술은 적은 출혈 양, 수술 후 적은 통증, 병원 재원기간 감소, 빠른 회복 기간 및 요실금이나 발기 부전 등의 합병증을 획기적으로 줄일 수 있는 것으로 주목 받고 있습니다. 그러나, 이러한 수술법에서는 수술 중 시야를 좋게 하기 위해 환자들은 수술 중의 머리가 아래로 행하며 다리가 위로 향하게 되는 30도의 경사가 심한 기복 상태를 취하여 수술을 받게 되고 수술 중 복강경 사용을 위해 기복 상태를 유지하는 것이 통상적입니다. 이러한 자세와 기복 상태는 수술 중 안압 증가와 밀접한 관계가 있음이 여러 연구들을 통해 보고되어 왔으며 안압 증가가 원인이 되어 매우 드물지만 허혈성 시신경증 같은 심각한 안과적 합병증을 발생 시킬 수 있다고 생각되고 있습니다. 근치적 전립선 절제술을 받는 환자 군의 특성상 고령이 많으며 여러 가지 질환을 동반할 가능성이 높고 수술 전 안과적 질병을 가진 환자도 증가함에 따라 로봇 전립선 절제술 후 안 손상과 같은 합병증이 발생할 위험이 더 증가할 것으로 생각됩니다. 따라서 이러한 환자들에 있어서 수술 중 안압 증가를 감소시키기 위한 방법을 모색하는 것이 수술 후 안과적 합병증을 줄이기 위해 중요하다고 생각됩니다.

이러한 복강경 수술에서는 수술하기 쉬운 환경은 근이완의 정도에 직접적인 영향을 받고 있습니다. 수술을 위한 전신 마취의 중요 요소에는 무통과 무의식, 기억상실과 함께 근육의 마비, 즉 근이완이 있습니다. 근이완제의 사용은 수술을 하는 외과의사에게 최적의 수술 환경을 제공하면서 마취와 수술 기술의 발전에 큰 역할을 하였습니다. 하지만 수술 중의 충분한 근이완 못지 않게 중요한 것은 수술이 끝나고 난 뒤의 완전한 가역입니다. 만약 근이완이 불충분하게 가역이 된다면 마취에서 깨어난 뒤에도 근력이 회복되지 않아 회복시간이 길어지고 호흡기계의 합병증의 위험이 증가하게 됩니다. 수술이 끝난 뒤 근이완제의 효과를 가역시키기 위해서 현재 표준 약제로 사용되는 약물은 네오스티그민 이라는 약제입니다. 이 약제는 항콜린에스테라아제의 하나로, 대부분의 환자에서 안전하고 효과적인 것으로 알려져 있어 전세계적으로 많은 마취의들이 이 약제를 근이완 가역제로 사용하고 있습니다. 하지만 수술이 끝난 시점에서 환자마다 근이완이 되어 있는 정도가 다를 수 있으며, 경우에 따라 근이완의 정도가 매우 깊거나 작용 시간이 긴 근이완제를 투여 받은 환자에서는 네오스티그민으로 충분한 가역을 보장할 수 없는 위험성이 있습니다. 최근에는 이러한 깊은 근이완 시에도 신속하게 근이완을 역전시킬 수 있는 슈감마덱스(sugammadex)가 개발이 되어 사용되고 있습니다.

본 연구의 목적은 50세 이상의 다빈치 로봇 전립선 절제술을 시행 받는 분들을 대상으로 현재까지 사용되고 있던 방법인 수술 중 중등도의 근이완 상태와 새로이 시행하려고 하는 깊은 근이완 상태로 무작위 배정하여 수술 중 깊은 근이완 상태를 유지함으로써 낮은 복강 내 압력으로도 충분한 시야와 수술하기 용이한 환경을 유도할 수 있으며 이로 인한 안압의 증감에 어떠한 영향을 미치는지 알아봄으로써, 다빈치 로봇 전립선 절제술 중 안압 증가를 감소시키며 안손상과 같은 합병증을 예방하는데 기여하고자 하는 것입니다.

**3. 실시기관, 대상자, 연구기간 및 연구책임자**

① 실시기관 : 연세대학교 의과대학 신촌세브란스병원

② 연구책임자 : 마취통증의학교실 교수 배선준 (원내 2228-2418)

연구담당자 : 마취통증의학교실 교수 유영철 (원내 2227-4643)

연구담당자 : 마취통증의학교실 임상조교수 김나영 (원내 2227-3549)

③ 대상환자 : 로봇 근치적 전립선절제술을 받는 500세 이상 80세 미만의 환자 68명을 대상

④ 연구기간 : IRB 통과 후 – 15개월

**4. 연구 방법에 대한 설명**

이 연구는 무작위 배정하에 중등도로만 근이완을 시키는 환자 군과 깊은 정도로 근이완을 시키는 환자군으로 나누어져 있으나, 그 이외의 모든 마취 방법은 군간 차이 없이 진행이 됩니다.

환자분이 수술실에 입실하시면, 심전도, 혈압계, 산소포화도와 마취 깊이 감시기 (BIS)를 부착한 뒤 전신 마취를 유도하게 됩니다. 전신 마취의 유도와 유지는 통상적으로 사용되는 진정제와 진통제로 이루어지고 마취 깊이가 적절하게 유지되도록 약제 농도는 조절됩니다. 전신 마취가 시작되고 환자분이 의식을 잃고 난 뒤에 근이완 감시 장치를 환자분의 손목의 척골신경을 따라 부착하여 근이완 정도를 지속적으로 관찰합니다. 신경근 이완의 감시는 근육의 이완을 필요로 하는 전신마취 중 통상적으로 사용되는 방법으로 acceleromyography라는 장치를 사용하게 됩니다. 환자의 의식이 소실되면, 무작위로 배정된 군에 따라서 다른 종류의 근이완제를 체중에 따라 조절하여 환자의 정맥으로 주입합니다. 근이완제에 의해 근육이 충분히 이완되었음을 확인한 후, 기관내관을 환자의 기관 내로 삽입합니다. 마취 직전, 마취 후 5분 뒤, 기복상태 유발 5분 후, 머리가 아래로 내려가는 자세를 취한 30분 후, 60분 후, 기복 상태 해제 후 다시 수평 자세 취한 5분 후, 튜브 발관 5분 후, 튜브 발관 30분 후 회복실에서, 튜브 발관 60분 후 회복실에서 환자의 안압과 혈압을 비롯한 혈역학적인 변화를 측정합니다.

근이완의 감시를 위해 본 연구에서 사용하는 전기 자극 방식은 두 가지 입니다. 첫 번째로 사연속자극 (Train-of four stimulation, TOF)이 있는데 이는 4번의 연속적인 전기 자극을 주고 이에 대한 수축 반응의 정도로 근이완의 회복 정도와 잔류 근이완 여부를 평가하는 방법입니다. 두 번째로는 강력한 강직성 자극을 준 후 다시 15번의 단일 자극을 연속적으로 주는 방법으로 (posttetanic count, PTC), TOF로는 가늠이 어려운 정도의 깊은 근이완 상태의 평가에 사용하며 본 연구에서는 깊은 근이완 상태로부터의 회복을 보고자 하기 때문에 이의 감시를 위해 PTC 자극을 사용합니다. 전신마취 하에 근이완제를 투여 받는 모든 환자에서 이러한 근이완 감시장치를 사용하도록 권장되고 있으며, 수술 중의 적절한 근이완과 회복기의 확실한 가역을 확인할 수 있어 통상적으로 사용되고 있는 감시 방법입니다. 근이완의 정도 감시를 위해 전기 자극을 주게 되나 이때 환자분은 전신 마취 상태이므로 통증이나 자극은 전혀 느끼지 않습니다. 수술 중의 근이완에는 로큐로니움과 아트라큐리움이 사용되며 가장 많이 쓰는 근이완제들입니다. 수술이 끝날 때까지 환자분의 마취 깊이와 근이완 정도를 일정 시간 간격으로 관찰하며 깊은 근이완 상태가 유지되도록 필요시에 근이완제가 추가 투여될 수 있습니다.

기복 상태 해제가 끝나면 지속 주입하던 근이완제를 중단하고 수술이 종료되고 근이완의 가역이필요한 시점에서 근이완의 정도를 확인한 후 무작위로 배정된 군에 따라서 네오스티그민 혹은 슈감마덱스를 정해진 용량만큼 정맥으로 투여합니다. 네오스티그민은 그 안전역이 넓고 효과적이어서 근이완의 회복을 위해 통상적으로 사용되어 온 표준 약제이나, 깊은 근이완 시에는 충분한 가역을 보장하기가 어렵습니다. 슈감마덱스는 최근 개발된 약제로서, 근이완제인 로큐로니엄을 포획하여 제거함으로써 깊은 근이완 상태를 유지하더라도 5분 이내에 신속하게 근이완의 가역이 가능한 길항제이며, 유럽과 호주 등지에서 널리 사용되면서 표준 근이완 가역제인 네오스티그민과 동등한 정도의 안전성이 입증되었습니다.

이와 같이 각 군에 따른 가역제가 투여된 후 환자가 근이완으로부터 충분히 회복된 것을 확인하고 기관내 발관을 시행한 뒤 회복실로 이송합니다. 회복실에서 간호사가 환자의 의식상태 및 근이완 상태를 평가하고, 이 외에도 오심 및 구토, 구강 건조 등을 확인합니다.

**본 연구에서 사용되는 근이완의 깊이에 따른 연축 반응의 예시.*


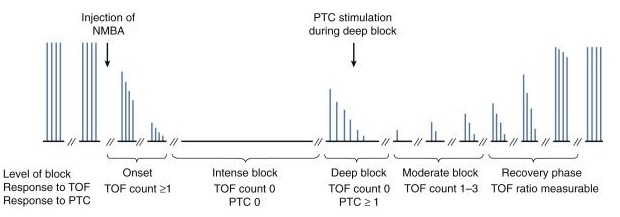


그림. 근이완제의 투여 후 TOF와 PTC를 이용한 근이완 깊이의 감시. 근이완의 깊이에 따라 자극에 대한 연축 반응이 다르게 나타난다.

**5. 예견되는 효과**

수술 중 깊은 근이완 상태를 유지함으로써 보다 낮은 복강 내 압력으로도 충분한 시야와 수술하기 용이한 환경을 유도할 수 있으며 이로 인한 안압의 감소 정도를 비교 관찰하여 효과적으로 안압의 증가를 막을 수 있어 수술 후 안 손상과 같은 합병증을 예방하는데 기여할 것으로 생각됩니다.

**6. 예상되는 위험성**

네오스티그민의 부작용은 서맥, 타액 분비 과다, 구역 및 구토, 어지러움, 과민 반응 등이 있고, 슈감마덱스의 부작용은 기침, 구강 건조, 빈맥 또는 서맥, 구토와 저혈압 이나, 모두 경미한 정도이며 보존적 치료로 회복이 되는 것들입니다. 네오스티그민은 표준 근이완 가역제로 전세계 마취의들이 사용해온 근이완 가역제이며 슈감마덱스는 현재 약 10년간 사용되면서 심각한 부작용이 발생한 경우가 매우 드문 약제이며 그 안전역이 넓어 네오스티그민과 동등한 정도의 안전성이 입증되었습니다. 연구에 참여하시는 분들은 회복기 동안 마취과 의사의 실시간 감시를 받게 되며 부작용이 발생하였을 경우 적절한 치료 약제 및 기구가 준비되어 있어 안전한 회복이 가능합니다.

**7. 대체 치료 방법**

근이완으로부터의 회복을 위해 네오스티그민을 투여하는 방법이 있으며 이는 통상적으로 사용되는 표준 약제입니다.

**8. 새로운 정보 제공 여부**

연구를 진행하는 중에 설명문에 언급되지 않았으나 새로 대상자가 알아야 할 정보가 있는 경우에는 알려드립니다.

**9. 보험 및 보상**

이 연구에서 사용되는 근이완 가역제인 네오스티그민 과 슈감마덱스의 약제비는 연구자가 부담하며, 이외의 감시 장치와 마취 약제는 전신 마취 시 통상적으로 사용되는 것으로만 진행하기 때문에, 연구를 위해 환자분에게 추가적으로 부과되는 검사, 방문 비용 등은 일체 없습니다.

**10. 대상자가 준수해야 하는 사항**

환자분께서는 전신 마취를 위해 지켜져야 하는 기본적인 사항만 잘 지켜주시면 됩니다.

**11. 대상자의 비밀 보장**

본 연구에 참여하는 대상자의 비밀은 보장되며, 연구의 결과가 출판될 경우 대상자의 신원은 비밀로 유지될 것입니다. 또한 타 연구 및 타 연구자(제3자)에게 대상자의 개인 정보가 노출되지 않을 것입니다. 제공모니터요원, 점검을 실시하는 자, 심사위원회 및 식품의약품안전청장은 대상자의 비밀보장을 침해하지 않고 관련규정이 정하는 범위 안에서 임상연구의 실시 절차와 자료의 신뢰성을 검증하기 위해 대상자의 의무기록을 직접 열람할 수 있으며 동의서 서식에 서명함으로써 대상자 또는 대리인이 이러한 자료의 직접열람을 허용하는 것을 포함합니다. 설명문 및 동의서 사본 1부를 받게 될 것이며 이 연구의 진행에 있어 의문점이 있으면 언제든 연구자에게 문의하시기 바랍니다. (연구자 유영철/김나영 연락처: 원내 2227-3549, 핸드폰 010-9990-8194) 대상자의 권익과 관련하여 추가적인 정보를 얻고자 할 경우 세브란스병원 연구 심의위원회 2228-0430~4 또는 세브란스병원 임상연구보호센터 2228-0451~4으로 연락하실 수 있습니다.

**14. 연구 참여/철회의 자발성**

본 임상연구에 대한 대상자의 임상연구 참여 여부 결정은 자발적인 것이며, 대상자가 원래 받을 수 있는 이익에 대한 손실이 없이도 임상연구에의 참여를 거부하거나 또는 임상연구 도중 언제라도 중도에 참여를 포기하실 수 있습니다. 연구를 거부하시더라도 안전한 수술을 위한 전신마취와 회복에는 영향이 없습니다.

**15. 임상 연구 참여가 중지되는 경우**

마취 전문의의 판단에 의하여 근이완 감시 장치의 적용이 어려워 근이완 깊이의 평가가 어려운 환자분은 연구 참여가 중지됩니다.

**16. 대상자 설명문 및 동의서 사본 1부는 대상자가 받게 됩니다. 본 연구는 세브란스병원 연구심의위원회에서 위험 및 이익을 분석하였으며 검토 후 승인된 연구입니다.**

**17. 연구책임자 및 담당자 연락처**

본 연구와 관련하여 24시간 연결 가능한 긴급 연락처로는 마취통증의학과 김나영 임상조교수 010-9990-8194 입니다

본 연구에 대한 문의사항, 염려, 불만 사항이 있으시면 아래로 연락하여 주십시오

연구책임자 : 배선준 교수 02)2227-3834, 010-7456-1311

연구담당자 : 유영철 교수 02)2227-4643, 010-8907-6937

연구담당자: 김나영 임상조교수 02)2227-3549, 010-9990-8194

대상자로서 귀하의 권리에 대하여 질문이 있는 경우에는 연구자에게 말씀하시거나 다음의 번호로 문의하실 수 있습니다.

세브란스 병원 임상연구심의위원회: 02)2228-0430~4

세브란스 병원 임상연구보호센터: 02)2228-0451~4

**대상자 동의서**

연구제목: 다빈치 로봇을 이용한 복강경적 근치적 전립선 절제술을 시행 받는 환자에서 수술 중 깊은 근이완을 통한 수술 시야 확보가 안압의 변화에 미치는 영향

|  |  | | | | | |
| --- | --- | --- | --- | --- | --- | --- |
| □ | 본인은 이 설명문을 읽었으며, 본 임상연구의 목적, 방법, 기대효과, 가능한 위험성, 타 치료 방법의 유무 및 내용, 건강 정보 관리 등에 대한 충분한 설명을 듣고 이해하였습니다. | | | | | |
| □ | 모든 궁금한 사항에 대해 질문하였고, 충분한 답변을 들었습니다. | | | | | |
| □ | 본 연구에 동의한 경우라도 언제든지 철회할 수 있고, 철회 이후 다른 적절한 치료를 받을 수 있음을 확인하였습니다. | | | | | |
| □ | 본인은 작성된 동의서 사본 1부를 받았음을 확인합니다. | | | | | |
| □ | 충분한 시간을 갖고 생각한 결과, 본인은 이 연구에 참여하기를 자유로운 의사에 따라 동의합니다. | | | | | |
|  | | | | | |  |
| 연구 참여자의 이름 | |  | 서명 |  | 날짜 |  |
|  | | | | | |  |
| 법정대리인 이름(해당되는경우) | |  | 서명 |  | 날짜 |  |
|  | | | | | |  |
| 입회자 이름 (해당되는 경우) | |  | 서명 |  | 날짜 |  |
|  | | | | | |  |
| 동의를 얻은 연구자의 이름 | |  | 서명 |  | 날짜 |  |
