# Supplementary material for: The Intraocular Pressure under Deep versus Moderate Neuromuscular Blockade during Low-Pressure Robot Assisted Laparoscopic Radical Prostatectomy in a Randomized Trial
Source: PLoS One. 2015 Aug 28;10(8):e0135412. doi: 10.1371/journal.pone.0135412 (PMC4552736; doi:10.1371/journal.pone.0135412)
Supplement: S1 Protocol — (DOCX) [file pone.0135412.s004.docx]

**Clinical Research Protocols**

1. Title

The impacts of surgical visibility through deep neuromuscular blockade on intraocular pressure in patients undergoing Robot-Assisted Laparoscopic Radical Prostatectomy –Randomized blind study-

1. Background

The prevalence of prostate cancer is extremely high in males worldwide.[[1](#_ENREF_1)] There are several surgical procedures in prostate cancer, inter alia Robot-Assisted Laparoscopic Radical Prostatectomy (RALRP) is the most frequently used and advanced surgical method for prostate cancer in the current practice.[[2](#_ENREF_2)] RALRP is being noticable for its less bleeding, less postoperative pain, short hospital stay, fast recovery, reduction in complications like incontinence and erectile dysfunction.[[3](#_ENREF_3)] However, during RALRP, patients should be placed in deep head-down (trendelenburg) position at 30 degrees with pneumoperitoneum for long time to achieve better surgical access.[[3](#_ENREF_3)] This position may be enough to cause severe ophthalmic damage such as ischemic optic neuropathy. Although the exact mechanism has not been identified, Trendelenburg position is being considered as a main cause of the intraocular pressure increase (IOP).[[2](#_ENREF_2),[4](#_ENREF_4)] Laparoscopic surgery is performed with intraperitoneal CO_2_ insufflation, which leads to a typical state of increased IOP. RALRP requires a steep Trendelenburg position and often higher insufflation pressure for optimal surgical condition, which will worsen the effect of increased IOP during surgery. [[2](#_ENREF_2),[5](#_ENREF_5)] Recently, serious optic damages according to this trendelenburg position after robotic or laparoscopic assisted radical prostatectomy have been reported. [[6](#_ENREF_6)] Also, as the nature of patients undergoing RALRP are mainly elderly and the patients with ocular disease which are not diagnosed preoperatively increases, it is important to make a strategy which reduces the IOP increase during surgery in order to prevent the occurrence of postoperative ophthalmic complications. Especially, RALRP requires often higher insufflation pressure (15-20mmHg) for optimal surgical condition. This CO_2_ pneumoperitoneum have known to increase peak inspiratory pressure (PIP), central venous pressure, end tidal CO2, transperitoneal absorption of CO2, and catecholamine release [[7](#_ENREF_7)], which will worsen the effect of increased IOP during surgery. [[2](#_ENREF_2),[8-15](#_ENREF_8)] Meanwhile, surgical condition in laparoscopic surgery are largely determined by depth of neuromuscular blockage,[[16](#_ENREF_16)] especially in procedure confined to a narrowing working space such as RALRP.

However, deep neuromuscular blockage is practically difficult due to several problems such as residual neuromuscular blockage and acetylcholine induced muscarinic receptor stimulation. With the introduction of suggamadex, rapid reversal of deep neuromuscular blockage is feasible. [[17](#_ENREF_17)] We hypothesize that continuous deep neuromuscular block during surgery improve surgical condition which enable RALRP performed using lower intra-abdominal pressure, and lower intra-abdominal pressure minimize these aforementioned factors increasing IOP during surgery.

3. Aim

To compare IOP at 60 minutes after being positioned in the ST position under CO_2_ pneumoperitoneum between moderate neuromuscular blockade group and deep neuromuscular blockade group.

4. Institution and study period

1) Institution: Yonsei University College of Medicine, Severance Hospital

2) Study period: Twenty four months from the approval of the IRB

5. Inclusion and exclusion criteria

1) Inclusion criteria: Patients with an American Society of Anesthesiologist (ASA) grade of I or II and aged 50–80 years who were scheduled to undergo elective RALRP visited the Anesthesiology preoperative evaluation clinic, and were enrolled after they provided written informed consent.

2) Exclusion criteria: Patients who had undergone previous ophthalmic surgery or were taking medications for glaucoma, those with current ophthalmic disease (glaucoma, diabetic retinopathy, cataract, and retinal detachment), and those with a baseline IOP of >30 mmHg were excluded. Patients with a history of allergy to sugammadex or neuromuscular blocking agents, known or suspected neuromuscular diseases, past history of retroperitoneal surgery, hypersensitivity to anesthetic agents, uncontrolled hypertension, liver or kidney disease, previous or familial history of malignant hyperthermia, medications that interact with muscle relaxants (anticonvulsants, certain antibiotics, magnesium, etc.), a body mass index (BMI) of 30 kg/m^2^, neurological or psychiatric illness, and mental retardation, as well as those incapable of reading the consent form because of illiteracy or language barriers, were excluded.

3)Screening: Past history (previous ophthalmic disease: glaucoma, cataract, retinal detachment, diabetic retinopathy), hemodynamic data (blood pressure, respiratory rate, heart rate), preoperative laboratory test for general anesthesia including blood test, coagulation test, routine chemistry test, x-ray, EKG, urine analysis

4) Withdrawal

Patients who wish to withdraw from the study can withdraw at any time. When the patients wish to withdraw, investigators may contact them for asking a reason for withdrawal. If the reasons for withdrawal are adverse events or abnormal laboratory tests, they should be recorded in the clinical research form.

6. Sample size calculation

In our previous study, IOP of the propofol TIVA group at pneumoperitoneum with trendelenberg position was 19.9±3.8 mmHg compared to 23.5±4.3mmHg of the sevoflurane inhalational anesthesia group.[[18](#_ENREF_18)] To detect a 3.6 mmHg difference in IOP (standard deviation of 4.3), power estimation analysis suggested that 31 patients per group would be required to obtain a power of 90%, considering a type I error of 0.05. Considering a drop-out rate of 10%, we recruited 34 patients in each group.

7. Study design & Methods

Patients with an American Society of Anesthesiologist (ASA) grade of I or II and aged 50–80 years who were scheduled to undergo elective RALRP visited the Anesthesiology preoperative evaluation clinic, and were enrolled after they provided written informed consent. After enrollment, patients were randomly allocated to either deep NMB Group (Group D, n= 34) or moderate NMB group (Group M, n=32) according to predetermined randomization sequence, which was generated in www.random.org with no dividing blocks and was covered up in a sealed envelope. Patients were administered 0.05 mg/kg of intramuscular midazolam as premedication. On arrival in the operating room, routine monitoring of noninvasive arterial blood pressure, electrocardiogram (ECG), oxygen saturation (SpO_2_), and bispectral index (BIS) (Aspect A-2000^®^; Aspect Medical System Inc., Newton, MA) were applied to the patient. Following the induction of general anesthesia with propofol (2 mg/kg) and remifentanil infusion (0.05–0.1 µg/kg/min), the radial artery was catheterized for continuous monitoring of arterial blood pressure and repetition of blood gas analysis. Mechanical ventilation was applied with a tidal volume of 8 mL/kg ideal body weight in 50% oxygen with air, a positive end-expiratory pressure of 5 cmH_2_O, and an inspiratory time:expiratory time ratio of 1:2. The respiratory rate was adjusted to 10–20 breaths/min to maintain the end-tidal CO_2_ tension (ETCO_2_) at 35–42 mmHg. The maintenance of anesthesia was undergone with sevoflurane (0.6–2.3 age-adjusted minimal alveolar concentration) and remifentanil (0.03–0.1 µg/kg/min) to target BIS scores of 40 to 60. Neuromuscular monitoring was performed using accelomyography (TOF-Watch SX^®^, Organon Ltd, Ireland) of the corrugator supercilli (CS) muscle. NMB agents (rocuronium or atracurium) were administered following calibration and stabilization of the train of four (TOF)-Watch. The patients were randomly allocated to one of two groups. Group D (deep NMB group) included patients who received an intravenous (IV) rocuronium bolus (1.0 mg/kg) following the continuous infusion of 0.6 mg/kg until the end of the ST position. Dose titration was assigned to an attending anesthetist via regulation of the bolus infusion speed to maintain a post-tetanic count (PTC) of 1 to 2. Sugammadex was administered to reverse the effects of NMB after surgery. Group M (moderate NMB group) included patients who received an IV atracurium bolus (0.4 mg/kg) following the continuous infusion of 0.1 mg/kg until the end of the ST position. Dose titration was assigned to an attending anesthetist via regulation of the bolus infusion speed to maintain a TOF count of 1 to 2. Neostigmine was used to reverse the effects of NMB after surgery. TOF was assessed every 15 min, and PTC was assessed if TOF was 0. NMB was maintained from induction until the end of the ST position. The patient was extubated once consciousness was regained and the TOF ratio was >0.9. After extubation, the patients were monitored for a minimum of 60 min in the post-anaesthetic care unit (PACU). Pneumoperitoneum was induced with CO_2_ insufflation of 20 mmHg. Following the insertion of trocars, an IAP of 8 mmHg was set from the previous 20 mmHg. All RALRP procedures were performed by a single experienced surgeon. Topical anesthetic eye drops (0.5% proparacaine HCl; Alcon, Seoul, Korea) were given to the patients in both groups. One blinded ophthalmologist measured IOP in all patients using the Tono-Pen XL handheld tonometer (Medtronic, Jacksonville, FL), three times at nine separate time points as follows: Before anaesthesia induction (awake in the supine position) (T0), 5 minutes after anaesthesia induction in the supine position (T1), 5 minutes after CO_2_ pneumoperitoneum in the supine position (T2), 30 minutes after CO_2_ pneumoperitoneum in the ST position (T3), 60 minutes after CO_2_ pneumoperitoneum in the ST position (T4), 5 minutes after returning to the horizontal position with desufflation of CO_2_ (T5), 5 minutes after tracheal extubation (T6), 30 minutes after tracheal extubation in the recovery room (T7), and 60 minutes after tracheal extubation in the recovery room (T8). The median value of the three IOP measurements was analysed for the data. At the end of the ST position, the surgeon was asked to rate the overall surgical conditions and worst surgical conditions using the 5-point rating scale as previously described. In addition, postoperative pain was assessed using a verbal Numerical Rating Scale for pain by blinded recovery nurses. Any postoperative respiratory events or known unfavourable events such as recurarization, dry mouth, nausea and vomiting, abdominal discomfort, headache were monitored.

8. Variables

Time points as below.

| Time | Event |
| --- | --- |
| T0 | Before anesthesia induction (awake in supine, horizontal position) |
| T1 | 5 min after anesthesia induction (mechanically ventilated, before CO_2_ pneumoperitoneum in supine, horizontal position) |
| T2 | 5 min after establishing CO_2_ pneumoperitoneum in horizontal position |
| T3 | 30 min after CO_2_ pneumoperitoneum with steep Trendelenburg position |
| T4 | 60 min after CO_2_ pneumoperitoneum with steep Trendelenburg position |
| T5 | 5 min after returning to horizontal position with desufflation of CO_2_ |
| T6 | 5 min after tracheal extubation in the operating room |
| T7  T8 | 30 min after tracheal extubation in the recovery room  60 min after tracheal extubation in the recovery room |

-Record the patient’s age, height, weight, ASA Class, Co-morbidity, duration of surgery, duration of anesthesia

-Record the total amount of rocuronium/atracurium during surgery

- Record the amount of neostigmine or sugammadex

- Record the total ephedrine amounts

- Record the variables as follow: Systolic, diastolic blood pressure, mean blood pressure, heart rate, spo2, intraocular pressure, BIS (T0-T8)

-Record the endtidal sevoflurane concentration, remifentanil concentration, peak airway pressure, minute ventilation, tidal volume, respiratory rate, temperature (T1-T5)

-Record arterial blood gas analysis (T1, T3, T5)

-Record intraabdominal pressure (T2, T3, T4)

-Record the total CO _2_ amount

-Surgeon was asked to rate the surgical condition (1: extremely poor, 2: poor, 3: acceptable, 4: good, 5: optimal)

- worst surgical conditions
- overall surgical conditions

- Record the period that intra-peritoneal pressure over 8mmHg

- Record the time and pressure when and how much IAP was increased.

- Record the time to reach TOF ratio 0.9

- After surgery, postoperative recovery variables observe as below:

- Clinical evidence of residual neuromuscular blockade (respiratory depression)
- Recularization event
- Mental status (awake and oriented, arousable with minimal stimulation, responsible only to tactile stimulation)
- Muscle strength (0-10 scale, 0: total paralysis, 10: normal muscle strength)
- 5 second head lift test
- Nausea (none, mild, moderate, severe)
- Vomiting
- Dry mouth

- Record every postoperative ocular or other complication and events.

9. End points

1) Primary endpoint

- comparison of the IOP at 60 minutes after being positioned in the ST position under CO_2_ pneumoperitoneum between moderate neuromuscular blockade group and deep neuromuscular blockade group

2) Secondary endpoints

- Comparison of the overall trends in IOP changes

- Surgical condition ratings given by the surgeon

- Comparison of the overall trends in IAP changes

- Correlation between IOP and IAP during pneumoperitoneum in both groups

10. Ethics and regulation

1) This study protocol conformed to the ethical guidelines of the 1975 Helsinki Declaration and International Conference on Harmonisation of Technical Requirements of Pharmaceuticals for Human Use (ICH) Note for Guidance on Good Clinical Practice (ICH, Topic E6, 1995)

2) This study was approved by the Institutional Review Board of Severance Hospital.

3) Compensation

There is no financial compensation for patients who participate in this study.

11. References

1. Hsing AW, Tsao L, Devesa SS. International trends and patterns of prostate cancer incidence and mortality. Int J Cancer. 2000;85: 60-67.

2. Awad H, Santilli S, Ohr M, Roth A, Yan W, Fernandez S, et al. The effects of steep trendelenburg positioning on intraocular pressure during robotic radical prostatectomy. Anesth Analg. 2009;109: 473-478.

3. Phong SV, Koh LK. Anaesthesia for robotic-assisted radical prostatectomy: considerations for laparoscopy in the Trendelenburg position. Anaesth Intensive Care. 2007;35: 281-285.

4. Rupp-Montpetit K, Moody ML. Visual loss as a complication of non-ophthalmic surgery: a review of the literature. Insight. 2005;30: 10-17.

5. Sugata A, Hayashi H, Kawaguchi M, Hasuwa K, Nomura Y, Furuya H. Changes in intraocular pressure during prone spine surgery under propofol and sevoflurane anesthesia. J Neurosurg Anesthesiol. 2012;24: 152-156.

6. Weber ED, Colyer MH, Lesser RL, Subramanian PS. Posterior ischemic optic neuropathy after minimally invasive prostatectomy. J Neuroophthalmol. 2007;27: 285-287.

7. Galizia G, Prizio G, Lieto E, Castellano P, Pelosio L, Imperatore V, et al. Hemodynamic and pulmonary changes during open, carbon dioxide pneumoperitoneum and abdominal wall-lifting cholecystectomy. A prospective, randomized study. Surg Endosc. 2001;15: 477-483.

8. Molloy BL. Implications for postoperative visual loss: steep trendelenburg position and effects on intraocular pressure. AANA J. 2011;79: 115-121.

9. Berg KT, Harrison AR, Lee MS. Perioperative visual loss in ocular and nonocular surgery. Clin Ophthalmol. 2010;4: 531-546.

10. Meininger D, Westphal K, Bremerich DH, Runkel H, Probst M, Zwissler B, et al. Effects of posture and prolonged pneumoperitoneum on hemodynamic parameters during laparoscopy. World J Surg. 2008;32: 1400-1405.

11. Mowafi HA, Al-Ghamdi A, Rushood A. Intraocular pressure changes during laparoscopy in patients anesthetized with propofol total intravenous anesthesia versus isoflurane inhaled anesthesia. Anesth Analg. 2003;97: 471-474, table of contents.

12. Grant GP, Szirth BC, Bennett HL, Huang SS, Thaker RS, Heary RF, et al. Effects of prone and reverse trendelenburg positioning on ocular parameters. Anesthesiology. 2010;112: 57-65.

13. Johnson DS, Crittenden DJ. Intraocular pressure and mechanical ventilation. Optom Vis Sci. 1993;70: 523-527.

14. Ismail SA, Bisher NA, Kandil HW, Mowafi HA, Atawia HA. Intraocular pressure and haemodynamic responses to insertion of the i-gel, laryngeal mask airway or endotracheal tube. Eur J Anaesthesiol. 2011;28: 443-448.

15. Lee LA, Roth S, Posner KL, Cheney FW, Caplan RA, Newman NJ, et al. The American Society of Anesthesiologists Postoperative Visual Loss Registry: analysis of 93 spine surgery cases with postoperative visual loss. Anesthesiology. 2006;105: 652-659; quiz 867-658.

16. Staehr-Rye AK, Rasmussen LS, Rosenberg J, Juul P, Gatke MR. Optimized surgical space during low-pressure laparoscopy with deep neuromuscular blockade. Dan Med J. 2013;60: A4579.

17. Martini CH, Boon M, Bevers RF, Aarts LP, Dahan A. Evaluation of surgical conditions during laparoscopic surgery in patients with moderate vs deep neuromuscular block. Br J Anaesth. 2013. 2013/11/19. doi: 10.1093/bja/aet377.

18. Yoo YC, Shin S, Choi EK, Kim CY, Choi YD, Bai SJ. Increase in intraocular pressure is less with propofol than with sevoflurane during laparoscopic surgery in the steep Trendelenburg position. Can J Anaesth. 2014;61: 322-329.

**The impact of surgical validity through profound neuromuscular blockade on intraocular pressure in patients undergoing robot assisted laparoscopic radical prostatectomy**

**Date** ___________ **Inicial** ___________ **serial number** ___________ **Group** _______

**Sex/Age** ___ / ___ **Ht/Wt** ___ / ___ **BMI**______ **BSA**______ **ASA class** ______

**Total duration of anesthesia** ____________ **Total duration of surgery** ___________

**PHx: HTN/DM** ( / ) **DM med** (PO / insulin ) **Others** ( )

**Previous ophthalmic disease** (glaucoma / cataract / retinal detachment / diabetic retinopathy / ophthalmic surgery/other :_____________)

**Total amounts of Vasopressor :** ephedrine mg, phenylephrine mg **Postoperative discharge day:**

**Total muscle relaxant amount** : ___/___, **Total reverse amount** : ___/___

**Intraoperative variables**

|  | T0 | T1 | T2 | T3 | T4 | T5 | T6 | T7 | T8 |
| --- | --- | --- | --- | --- | --- | --- | --- | --- | --- |
| BIS |  |  |  |  |  |  |  |  |  |
| IOP | / / | / / | / / | / / | / / | / / | / / | / / | / / |
| Abd pr |  |  |  |  |  |  |  |  |  |
| **ventilator** | | | | | | |  |  |  |
| EtCO_2_ |  |  |  |  |  |  |  |  |  |
| M/V |  |  |  |  |  |  |  |  |  |
| TV |  |  |  |  |  |  |  |  |  |
| RR |  |  |  |  |  |  |  |  |  |
| PAP |  |  |  |  |  |  |  |  |  |
| Remifentanil |  |  |  |  |  |  |  |  |  |
| Agent |  |  |  |  |  |  |  |  |  |
| **vital** | | | | | | |  |  |  |
| SpO_2_ |  |  |  |  |  |  |  |  |  |
| BP |  |  |  |  |  |  |  |  |  |
| PR |  |  |  |  |  |  |  |  |  |
| **ABGA** | | | | | | |  |  |  |
| PH |  |  |  |  |  |  |  |  |  |
| PaO_2_ |  |  |  |  |  |  |  |  |  |
| PaCO_2_ |  |  |  |  |  |  |  |  |  |
| HCO3^-^ |  |  |  |  |  |  |  |  |  |
| Lactate |  |  |  |  |  |  |  |  |  |

**M/V**: minute volume, **PAP** : Peak airway pressure, **TV**: tital volume, **Abd pr**: abdominal pressure

**Total I & O**

| Fluid (crystalloid) |  |
| --- | --- |
| Fluid (Colloid) |  |
| Transfusion (PRBC) |  |
| Urine output |  |
| Bleeding |  |

**Surgical condition rating**

| Worst surgical space condition |  |
| --- | --- |
| Overall surgical space condition |  |

**1: extremely poor, 2: poor, 3: acceptable, 4: good, 5: optimal**

Grade 5 (optimal), optimal surgical conditions; grade 4 (good), nonoptimal conditions, but an intervention is not required; grade 3 (acceptable), wide surgical view, but an intervention can improve surgical conditions, grade 2 (poor), inadequate conditions, there is a visible view, but an intervention is necessary to ensure acceptable surgical conditions; grade 1 (extremely poor), inability to perform surgery; therefore, intervention is necessary.

**Postoperative recovery variables**

| **Variables** | 30 minutes in PACU | 60 minutes in PACU |
| --- | --- | --- |
| **Clinical evidence of residual neuromuscular blockade (ex: respiratory depression)** |  |  |
| **Recularization event** |  |  |
| **Mental status *** |  |  |
| **Muscle strength *** |  |  |
| **5 second head tilt test (YES/NO)** |  |  |
| **Nausea (none/mild/moderate/severe)** |  |  |
| **Vomiting (YES/NO)** |  |  |
| **Dry mouth (none/mild/moderate/severe)** |  |  |

**Mental status *** awake and oriented, arousable with minimal stimulation, responsible only to tactile stimulation

**Muscle strength *** 0-10 scale, 0: total paralysis, 10: normal muscle strength
