# Supplementary material for: The Intraocular Pressure under Deep versus Moderate Neuromuscular Blockade during Low-Pressure Robot Assisted Laparoscopic Radical Prostatectomy in a Randomized Trial
Source: PLoS One. 2015 Aug 28;10(8):e0135412. doi: 10.1371/journal.pone.0135412 (PMC4552736; doi:10.1371/journal.pone.0135412)
Supplement: S2 Protocol — (DOCX) [file pone.0135412.s005.docx]

**임상 연구 계획서**

1. 제목

다빈치 로봇을 이용한 복강경적 근치적 전립선 절제술을 시행 받는 환자에서 수술 중 깊은 근이완을 통한 수술 시야 확보가 Intraocular Pressure의 변화에 미치는 영향 – 무작위 배정 연구 –

1. 연구 배경

전립선 암은 미국 내에서 남자들에게 진단되는 암중 가장 흔한 암중 하나로,[[1](#_ENREF_1)] 전립선 암의 수술법은 여러 방법이 있지만 그 중 다빈치 로봇을 이용한 전립선 절제술은 가장 최신이며 기술적으로 가장 진보한 수술법 중 하나이다.[[2](#_ENREF_2)] 다빈치 로봇을 이용한 전립선 절제술은 적은 출혈 양, 수술 후 적은 통증, 병원 재원 기간 감소, 빠른 회복 기간 및 요실금 이나 발기부전 등의 합병증을 획기적으로 줄일 수 있는 것으로 주목 받고 있다. [[3](#_ENREF_3)] 그러나 이러한 수술법에서는 수술 중 시야를 좋게 하기 위해 환자가 장시간 기복상태(pneumoperitoneum)를 유지하며 30도의 경사가 심한 trendelenburg 자세를 취하게 된다. [[3](#_ENREF_3)] 이러한 경사가 심한 Trendelenburg 자세는, 드물지만 ischemic optic neuropathy 같은 심각한 안손상을 유발할 수 있다. 이에 대한 정확한 기전은 밝혀지지 않았지만 trendelenburg 자세로 인한 안구내압 (intraocular pressure, IOP)의 증가가 중요한 원인으로 생각되고 있다.[[2](#_ENREF_2),[4](#_ENREF_4)] 최근 연구에서 trendelenburg 자세와 수술 중 기복 상태로 인해 증가된 호기말 이산화탄소 분압이 수술 중 IOP의 증가와 밀접한 관계가 있음이 보고 되었다.[[2](#_ENREF_2),[5](#_ENREF_5)] 최근 다빈치 로봇을 이용하거나 복강경을 이용해서 전립선 절제술을 받는 환자들이 증가하면서 수술 후 심각한 안손상을 경험한 사례가 여러 차례 보고되고 있다.[[6](#_ENREF_6)] 뿐만 아니라 전립선 절제 수술을 받는 환자 군의 특성상 고령이 많으며 여러 가지 질환을 동반할 가능성이 높고 수술 전 안과적 질병을 가진 환자도 증가함에 따라 다빈치 로봇 전립선 절제술 후 안손상과 같은 합병증이 발생할 위험이 증가할 것으로 생각 된다. 따라서 이러한 환자에 있어서 수술 중 IOP 증가를 감소시키기 위한 전략을 세우는 것이 수술 후 안과적 합병증을 줄이기 위해 매우 중요하다. 특히 다빈치 로봇을 이용한 전립선 절제술은 시야 확보와 수술하기 용이한 환경을 위하여 더 높은 복강내 압력(15-20mmHg)를 요구하는데, 이러한 이산화탄소를 이용한 기복상태의 유발은 최대 기도압(peak inspiratory pressure, PIP), 중심정맥압 (central venous pressure, CVP), 이산화탄소 분압과 카테콜라민 (catecholamine)의 분비를 증가시키며[[7](#_ENREF_7)], 이는 모두 수술 중 IOP를 증가시키는 중요한 요인으로 알려져 있다.[[2](#_ENREF_2),[8-15](#_ENREF_8)] 한편 복강경 수술에서 수술하기 용이한 환경은 근이완의 정도에 직접적인 영향을 받는다.[[16](#_ENREF_16)] 하지만 수술 중 깊은 근이완 상태를 유지하면 수술이 종료된 후 근이완이 회복 되기 위한 시간이 짧게는 수분에서 길게는 한 시간 이상 필요하며, 다빈치 로봇을 이용한 전립선 절제술을 시행 받는 고령의 환자에서는 수시간까지 연장될 수도 있다. 이러한 이유로 현재까지는 수술 중 중등도의 근이완을 유지할 수 밖에 없었다. 슈감마덱스(suggamadex)는 근이완제인 로큐로니엄(rocuronium)을 포획하여 완전히 제거함으로써 수술 중 깊은 근이완 상태를 유지하더라도 5분 이내에 근이완을 역전시킬 수 있음이 보고 되었다. [[17](#_ENREF_17)] 이에 본 연구자들은 다빈치 로봇 전립선 절제술을 시행 받는 환자들에서 수술 중 깊은 근이완 상태를 유지함으로써 보다 낮은 복강내 압력으로도 충분한 시야와 수술하기 용이한 환경을 유도할 수 있으며, 이로 인한 안압의 상승을 억제할 수 있다고 가정하였다.

3. 연구 목적

무작위로 배정된 통상적인 중등도 근이완 상태와 깊은 근이완 상태를 유지한 환자 군에서 충분한 시야와 수술하기 용이한 환경을 조성하기 위하여 필요한 기복상태 압력과 IOP를 비교한다.

4. 연구 수행장소 및 기간

1) 실시기관: 연세대학교 의과대학 신촌 세브란스 병원

2) 연구기간: IRB 승인 후 15개월

5. 대상자 선정과 제외기준

1)대상자 선정: 연구 참여에 동의한 50세 이상 80세 미만의 미국마취협회 신체기준 I, II인 계획된 다빈치 로봇을 이용한 전립선 절제술을 받는 환자를 대상으로 한다.

2)제외기준: 안과적 질환을 가지고 있던 환자 (glaucoma, diabetic retinopathy, cataract, retinal detachment), 안과적 수술을 받은 과거력을 가진 환자, Screening 검사 시 높은 IOP (above 30mHg)를 가진 환자, 심장질환 (unstable angina, congestive heart failure) 가진 환자, 조절되지 않는 고혈압을 가진 환자, 응고장애를 가진 환자, 중증의 신장애 환자, 천식 환자, 악성 고열증의 병력이 있는 환자, 간부전을 진단받았거나 의심되는 환자, 아편양 제제, 비탈분극성 근이완제 등 마취관련 약제에 알러지가 있는 환자, 근이완제와 상호작용을 하는 것으로 알려진 약제를 복용 중인 환자 (항경련제, certain antibiotics, magnesium 등), 비만인 환자 (BMI > 30kg/m^2^ )는 제외한다. 그리고 본 연구의 목적과 위험에 대한 설명을 충분히 이해한 후 동의할 수 없는 외국인과 문맹은 연구에서 제외한다.

3)스크리닝 검사 항목: 과거력(안과적 질병 : 녹내장, 백내장, 망막박리, 당뇨성 망막증) 및 병력, 혈역학적 지표 (혈압, 호흡수, 맥박수), 일반 혈액 및 혈액 응고 검사, 일반 화학 검사, 심전도, 흉부 엑스레이, 소변 검사 등 일반적으로 전신마취를 위해 시행하는 술 전 검사.

4)탈락 기준

환자는 어떠한 이유로라도 아무 때나 시험을 철회할 권리를 가진다. 시험자는 대상자 또는 대상자를 책임지는 가족과 접촉하여 철회 사유를 알아내도록 한다. 시험 철회가 이상 반응이나 검사 결과의 비정상으로 인한 것이라면 이에 대한 주요 반응이나 검사 결과가 CRF에 기록된다.

6. 목표 대상자의 수 및 산출 근거

최근 본 연구자들이 시행한 propofol과 sevoflurane의 IOP의 감소 정도를 비교한 연구에서[[18](#_ENREF_18)] 수술 중 가장 IOP가 증가한 시점 (30 min after Trendelenburg position with pneumoperitoneum)에서 propofol 군의 IOP는 19.9±3.8mmHg, 대조약인 sevoflurane을 이용한 마취 후 IOP는 23.5±4.3mmHg 이었다. 본 연구에서 군간 필요한 환자수는 alpha 0.05, 90% power에서 31명의 환자가 필요하다. 따라서 중도 탈락율을 10%로 고려하여 각 군당 34명씩 연구를 진행하도록 한다.

7. 연구 설계 및 방법

다빈치 로봇 전립선 절제술로 전신 마취과 계획된 미국 마취과 학회 신체 등급 분류 1-2 군에 해당하는 50세 이상의 성인 환자들을 대상으로 연구는 전향적으로 진행하며 우선 주치의에게 환자의 연구 참여에 대한 협조 및 승낙을 받는다. 인터넷 상의 컴퓨터 프로그램 (<http://www.randomizer.org>) 을 이용하여 무작위로 깊은 근이완 (deep neuromuscular block, DNB)군 혹은 중등도 근이완 (moderate neuromuscular block, MNB)군으로 군을 배정한다. 모든 환자는 전처치로 midazolam 0.05 mg/kg 을 근주 하고, 수술실에 입실하여 혈압, 심전도, 맥박산소포화도, BIS 를 측정하고 마취를 시작한다. 마취 유도는propofol과 remifentanil로 시행한다. 그리고 지속적인 동맥혈압의 측정과 혈액 가스 검사를 위해서 동맥관 삽입을 실시한다. 수술 중 기계 호흡은 호흡 용적은 50%의 산소에서 8 ml/kg, 5 cmH_2_O의 양압 환기와, 1:2 의 비율의 흡기와 호기 호흡으로 설정한다. 호흡 수는 10-20로 조절하는데 호기말 이산화탄소 농도가 35-45mmHg 범위 내에서 유지한다. 마취 유지 역시 sevoflurnage 과 remifentanil로 마취의 깊이는 Bispectral index (BIS) monitoring 을 사용하여 조절하며 BIS의 수치는 40~60 사이가 되도록 유지한다. 수술 중 근이완 감시는 근이완 감시 장치인 acceleromyography (TOF-Watch® SX, Roganon Ireland Ltd., a subsidiary of Merch and Co., Swords, Co. Dublin, Ireland)를 이용하여 corrugator supercilli (CS)에서 모니터 한다. acceleromyography의 Calibration의 전 과정이 완료되면 근이완제를 정주한다. 무작위 배정에 따라 정해진 군의 종류에 따라서 DNB 군에서는 Rocuronium을 1.0mg/kg 로 기관 삽관 후 15초 간격으로 지속적인 train of four (TOF) 자극을 주면서 TOF 연축 반응이 더 이상 나타나지 않는 것이 확인되면 이후 Posttetanic count (PTC) 자극을 15분 간격으로 주면서 PTC가 2개 이하로 유지되도록 한다. PTC 가 3이상 보일 때 마다 0.6mg/kg/hr 의 유지용량을 투여하여 연축 반응을 2개 이하로 유지하도록 한다. MNB 군에서는 Atracurium을 0.4mg/kg로 기관 삽관 후, TOF 가 2로 유지되도록 0.1mg/kg/hr 로 atracurium의 지속 정주를 한다. 두 군 모두 기복상태 종료 후 지속 정주 하던 근이완제를 정지시킨다. 마취 종료 시 근이완의 길항은 군 별로 다음과 같이 시행하며, 두 군 모두에서 TOF ratio 0.9 이상이 되는 시점에서 기관 발관을 시행한다. MNB 군에서는 기존의 근이완 역전 방식대로 TOF 자극에 대해 T2 반응이 나타난다면 네오스티그민을 glycopyrrolate 10 ㎍/kg와 함께 투여하고, DNB 군에서는 슈감마덱스를 투여 하여 근이완을 역전한다. 수술 중 intraperitoneal pressure는 Troca를 20mmHG 에서 뚫은 후 8mmHg 로 낮춘 뒤 수술자의 요구에 따라 단계적으로 1mmHg씩 올리도록 하며 어느 시점에서 얼마까지 올리는 지를 기록한다. 수술 방법은 두군 모두에서 기존의 수술 방법과 동일하게 한명의 경험이 많은 의사가 시행한다. 모든 환자의 IOP는 안압 측정기기 (Tono-Pen AVIA®)를 사용하여 한 연구자가 측정하며 안압을 재기 전에 국소 안약을 넣고 시행한다. 각 시점의 안압은 총 9회의 서로 다른 시점에서 3회 측정한 중간 값을 분석에 사용한다. 9시점은 다음과 같다. 이는 기관 삽관 후 기계 호흡 시작 5분 후 (T1), supine 상태에서 기복상태 유발 5분 후 (T2) trendelenburg 자세 취한 30분 후 (T3), trendelenburg 자세 취한 60분 후 (T4), 기복 상태 종료 후 trendelenburg 자세에서 supine 상태로 체위 전환 5분 후 (T5), 튜브 발관 후 5분뒤 수술방에서 (T6), 튜브 발관 후 30분 뒤 회복실에서 (T7), 튜브 발관 후 60분 뒤 회복실에서 (T8) 의 시점에서 실시한다. ST position 종료 시점에서는surgical condition 에 대한 rating 을 한다. 그래서 전반적인 수술의 만족도와 가장 안 좋았던 수술 컨디션에 대해서 5가지의 항목으로 평가한다. 환자가 회복실로 이송되면, 배정된 군을 모르는 회복실 간호사가 환자의 의식상태, 전신근육약화 여부와 근이완의 재발생 여부, 회복실에서 호흡 장애, 오심, 구토, 구강 건조 여부를 관찰한다.

**8.** 관찰항목

-연구 중 측정 시점은 다음 표와 같다.

| Time | Event |
| --- | --- |
| T0 | Before anesthesia induction (awake in supine, horizontal position) |
| T1 | 5 min after anesthesia induction (mechanically ventilated, before CO_2_ pneumoperitoneum in supine, horizontal position) |
| T2 | 5 min after establishing CO_2_ pneumoperitoneum in horizontal position |
| T3 | 30 min after CO_2_ pneumoperitoneum with steep Trendelenburg position |
| T4 | 60 min after CO_2_ pneumoperitoneum with steep Trendelenburg position |
| T5 | 5 min after returning to horizontal position with desufflation of CO_2_ |
| T6 | 5 min after tracheal extubation in the operating room |
| T7  T8 | 30 min after tracheal extubation in the recovery room  60 min after tracheal extubation in the recovery room |

-환자의 나이, 키, 몸무게, 과거력, 수술 시간, 마취시간을 기록한다.

-근이완제의 마취 중 총 사용량을 기록한다.

-네오스티그민 또는 슈감마덱스의 용량을 기록한다.

-마취 중 혈압 상승제 또는 혈압 하강제의 투여 및 종류 및 용량

-수축기 혈압, 이완기 혈압, 평균 혈압, 심박수, 산소 포화도, 안압, BIS 를 기록한다. (T0~T8 시점)

-Sevoflurane 의 호기말 농도, remifentanil의 효과처 농도, Peak air way pressure, 분당 -호흡량, 일회 호흡량, 분당 호흡수, 식도 내 체온을 기록한다. (T1 – T5시점)

-동맥혈 가스 분압 측정을 한다. (T1, T3, T5 시점)

-복강 내 압력을 기록 한다. (T2, T3, T4 시점)

-기복 상태 종료 시점에 총 들어간 이산화탄소의 양을 기록한다.

-수술자의 surgical condition rating (1: extremely poor, 2: poor, 3: acceptable, 4: good, 5: optimal)을 측정한다.

- 가장 나빴을 때의 surgical space conditions
- 전체적인 surgical space conditions

-전체 기복상태 기간 중 intra-peritoneal pressure가 8mmHg 이상이 필요했던 시간의 비율을 기록한다.

-수술 중 surgical condition 을 위하여 intra-abdominal pressure 를 조절한 시점과 정도를 기록한다.

- 기복상태 시작 후 ( ) min, ( ) mmHg 까지 올렸음.

- 슈감마덱스 or neostigmine 투여 이후 TOF ratio 0.9 에 도달하는 시간을 기록한다.

- 마취 종료 후 회복실에서 다음의 항목들을 15분마다 관찰한다.

- 잔류 근이완의 임상적 증거 (호흡곤란)
- 근이완의 재발생 여부
- 의식상태 (awake and oriented, arousable with minimal stimulation, responsible only to tactile stimulation)
- 전신 근육 약화 (0-10 scale, 0: total paralysis, 10: normal muscle strength)
- 5초 head lift test
- 오심 (none, mild, moderate, severe)
- 구토
- 구강 건조

- 모든 환자에 대하여 수술 후 퇴원 전까지 발생한 안과적 합병증을 관찰하고 기록한다.

9. 유효성 평가기준

1) 일차 목적

-깊은 근이완 군과 기복상태와 중등도 근이오나 군에서의 Trendelenburg 자세를 취한 60분 후의 IOP의 비교

2) 이차 목적

- Perioperative period 전반에 걸쳐서 측정 시점의 IOP의 비교

- Surgical condition rating 비교

- Perioperative period 전반에 걸쳐서 측정 시점의 기복상태 압력 비교

- IOP와 기복상태 압력의 Correlation 확인

10. 연구 윤리 및 규제

1) 본 연구는 The ethical guidelines of the 1975 Helsinki Declaration 과 International Conference on Harmonisation of Technical Requirements of Pharamceuticals for Human Use (ICH) Note for Guidance on Good Clinical Practice (ICH, Topic E6, 1995) 을 따른다.

2) 본 연구는 세브란스병원 연구윤리심의위원회에서 승인되었다.

3) 보상

본 연구 참여와 관련한 금전적인 보상은 없다.

11. 참고 문헌

1. Hsing AW, Tsao L, Devesa SS. International trends and patterns of prostate cancer incidence and mortality. Int J Cancer. 2000;85: 60-67.

2. Awad H, Santilli S, Ohr M, Roth A, Yan W, Fernandez S, et al. The effects of steep trendelenburg positioning on intraocular pressure during robotic radical prostatectomy. Anesth Analg. 2009;109: 473-478.

3. Phong SV, Koh LK. Anaesthesia for robotic-assisted radical prostatectomy: considerations for laparoscopy in the Trendelenburg position. Anaesth Intensive Care. 2007;35: 281-285.

4. Rupp-Montpetit K, Moody ML. Visual loss as a complication of non-ophthalmic surgery: a review of the literature. Insight. 2005;30: 10-17.

5. Sugata A, Hayashi H, Kawaguchi M, Hasuwa K, Nomura Y, Furuya H. Changes in intraocular pressure during prone spine surgery under propofol and sevoflurane anesthesia. J Neurosurg Anesthesiol. 2012;24: 152-156.

6. Weber ED, Colyer MH, Lesser RL, Subramanian PS. Posterior ischemic optic neuropathy after minimally invasive prostatectomy. J Neuroophthalmol. 2007;27: 285-287.

7. Galizia G, Prizio G, Lieto E, Castellano P, Pelosio L, Imperatore V, et al. Hemodynamic and pulmonary changes during open, carbon dioxide pneumoperitoneum and abdominal wall-lifting cholecystectomy. A prospective, randomized study. Surg Endosc. 2001;15: 477-483.

8. Molloy BL. Implications for postoperative visual loss: steep trendelenburg position and effects on intraocular pressure. AANA J. 2011;79: 115-121.

9. Berg KT, Harrison AR, Lee MS. Perioperative visual loss in ocular and nonocular surgery. Clin Ophthalmol. 2010;4: 531-546.

10. Meininger D, Westphal K, Bremerich DH, Runkel H, Probst M, Zwissler B, et al. Effects of posture and prolonged pneumoperitoneum on hemodynamic parameters during laparoscopy. World J Surg. 2008;32: 1400-1405.

11. Mowafi HA, Al-Ghamdi A, Rushood A. Intraocular pressure changes during laparoscopy in patients anesthetized with propofol total intravenous anesthesia versus isoflurane inhaled anesthesia. Anesth Analg. 2003;97: 471-474, table of contents.

12. Grant GP, Szirth BC, Bennett HL, Huang SS, Thaker RS, Heary RF, et al. Effects of prone and reverse trendelenburg positioning on ocular parameters. Anesthesiology. 2010;112: 57-65.

13. Johnson DS, Crittenden DJ. Intraocular pressure and mechanical ventilation. Optom Vis Sci. 1993;70: 523-527.

14. Ismail SA, Bisher NA, Kandil HW, Mowafi HA, Atawia HA. Intraocular pressure and haemodynamic responses to insertion of the i-gel, laryngeal mask airway or endotracheal tube. Eur J Anaesthesiol. 2011;28: 443-448.

15. Lee LA, Roth S, Posner KL, Cheney FW, Caplan RA, Newman NJ, et al. The American Society of Anesthesiologists Postoperative Visual Loss Registry: analysis of 93 spine surgery cases with postoperative visual loss. Anesthesiology. 2006;105: 652-659; quiz 867-658.

16. Staehr-Rye AK, Rasmussen LS, Rosenberg J, Juul P, Gatke MR. Optimized surgical space during low-pressure laparoscopy with deep neuromuscular blockade. Dan Med J. 2013;60: A4579.

17. Martini CH, Boon M, Bevers RF, Aarts LP, Dahan A. Evaluation of surgical conditions during laparoscopic surgery in patients with moderate vs deep neuromuscular block. Br J Anaesth. 2013. 2013/11/19. doi: 10.1093/bja/aet377.

18. Yoo YC, Shin S, Choi EK, Kim CY, Choi YD, Bai SJ. Increase in intraocular pressure is less with propofol than with sevoflurane during laparoscopic surgery in the steep Trendelenburg position. Can J Anaesth. 2014;61: 322-329.

**The impact of surgical validity through profound neuromuscular blockade on intraocular pressure in patients undergoing robot assisted laparoscopic radical prostatectomy**

**Date** ___________ **Inicial** ___________ **serial number** ___________ **Group** _______

**Sex/Age** ___ / ___ **Ht/Wt** ___ / ___ **BMI**______ **BSA**______ **ASA class** ______

**총 마취시간** ____________ **총** **수술시간** ___________

**PHx: HTN/DM** ( / ) **DM med** (PO / insulin ) **Others** ( )

**안과 질환 과거력** (녹내장/백내장/각막박리/당뇨성 망막증/안과적 수술여부_____________)

**Vasopressor 총 사용량**: ephedrine mg, phenylephrine mg **Postoperative discharge day:**

**Total muscle relaxant amount** : ___/___, **Total reverse amount** : ___/___

**Intraoperative variables**

|  | T0 | T1 | T2 | T3 | T4 | T5 | T6 | T7 | T8 |
| --- | --- | --- | --- | --- | --- | --- | --- | --- | --- |
| BIS |  |  |  |  |  |  |  |  |  |
| IOP | / / | / / | / / | / / | / / | / / | / / | / / | / / |
| Abd pr |  |  |  |  |  |  |  |  |  |
| **ventilator** | | | | | | |  |  |  |
| EtCO_2_ |  |  |  |  |  |  |  |  |  |
| M/V |  |  |  |  |  |  |  |  |  |
| TV |  |  |  |  |  |  |  |  |  |
| RR |  |  |  |  |  |  |  |  |  |
| PAP |  |  |  |  |  |  |  |  |  |
| Remifentanil |  |  |  |  |  |  |  |  |  |
| Agent |  |  |  |  |  |  |  |  |  |
| **vital** | | | | | | |  |  |  |
| SpO_2_ |  |  |  |  |  |  |  |  |  |
| BP |  |  |  |  |  |  |  |  |  |
| PR |  |  |  |  |  |  |  |  |  |
| **ABGA** | | | | | | |  |  |  |
| PH |  |  |  |  |  |  |  |  |  |
| PaO_2_ |  |  |  |  |  |  |  |  |  |
| PaCO_2_ |  |  |  |  |  |  |  |  |  |
| HCO3^-^ |  |  |  |  |  |  |  |  |  |
| Lactate |  |  |  |  |  |  |  |  |  |

**M/V**: minute volume, **PAP** : Peak airway pressure, **TV**: tital volume, **Abd pr**: abdominal pressure

**Total I & O**

| Fluid (crystalloid) |  |
| --- | --- |
| Fluid (Colloid) |  |
| Transfusion (PRBC) |  |
| Urine output |  |
| Bleeding |  |

**Surgical condition rating**

| Worst surgical space condition |  |
| --- | --- |
| Overall surgical space condition |  |

**1: extremely poor, 2: poor, 3: acceptable, 4: good, 5: optimal**

Grade 5 (optimal), optimal surgical conditions; grade 4 (good), nonoptimal conditions, but an intervention is not required; grade 3 (acceptable), wide surgical view, but an intervention can improve surgical conditions, grade 2 (poor), inadequate conditions, there is a visible view, but an intervention is necessary to ensure acceptable surgical conditions; grade 1 (extremely poor), inability to perform surgery; therefore, intervention is necessary.

**회복실 입실 후 평가사항**

| **회복실 입실 중 평가 사항** | 30분 | 60분 |
| --- | --- | --- |
| **잔류근이완의 임상적 근거(예:호흡곤란)** |  |  |
| **근이완의재발생여부(예:TOF:0.7)** |  |  |
| **의식상태*** |  |  |
| **전신근육약화 *** |  |  |
| **5초 head tilt test (YES/NO)** |  |  |
| **오심 (none/mild/moderate/severe)** |  |  |
| **구토 (YES/NO)** |  |  |
| **구강건조 (none/mild/moderate/severe)** |  |  |

**의식상태*** awake and oriented, arousable with minimal stimulation, responsible only to tactile stimulation

**전신근육약화 *** 0-10 scale, 0: total paralysis, 10: normal muscle strength
